# Attitude toward end-of-life care in emergency medicine residents- can a short workshop make a difference?

**DOI:** 10.1371/journal.pone.0280229

**Published:** 2023-01-11

**Authors:** Nader Sadigh, Javad Seyedhosseini, Mamak Tahmasebi, Farzaneh Shirani

**Affiliations:** 1 Department of Emergency Medicine, Shariati Hospital, Tehran University of Medical Sciences, Tehran, Iran; 2 Department of Emergency Medicine, Iran University of Medical Sciences, Tehran, Iran; 3 Radiotherapy/Oncology Department, Cancer Institute, Tehran University of Medical Sciences, Tehran, Iran; University of Hafr Al-Batin, SAUDI ARABIA

## Abstract

**Background:**

There is a growing demand for palliative care (PC) in Emergency departments (ED) as the number of patients who need end-of-life (EOL) care is increasing. Despite significant variability amongst residency programs, there is a lack of structured core curriculum for PC/EOL care in most emergency medicine (EM) training programs, which often do not meet the needs of EM physicians. In this study, we evaluate the effect of a short EOL care workshop on changing the attitude of Iranian EM residents towards EOL care in ED.

**Method:**

In this prospective before/after educational study at Tehran University of medical science, we enrolled 40 EM residents using a random sampling method. We obtained demographic and practice background information, and participants underwent a half-day PC training workshop designed by an expert panel. We administered a translated and validated Standard PEAS (physician End of Life Care Attitude Scale) questionnaire before and four weeks after an educational intervention. Baseline and differences in attitude were reported and compared by paired t-test, repeated measure ANOVA, and ANOVA.

**Results:**

None of the participants had prior experience of formal PC training. All of the 40 participants completed the follow-up questionnaire. Baseline attitude was not different among demographic groups. The mean (SD) PEAS score before and four weeks after the workshop was 86.9 (5.8) versus 89(6.9), respectively (P = 0.023). Residents with no previous close exposure to a terminal illness in their family members had significantly more attitude change than those with such an experience (P = 0.045).

**Conclusion:**

A brief educational intervention improved EM residents’ attitudes toward EOL care. The optimal design and characteristics of this educational intervention yet remain to be defined by further studies.

## Introduction

The aging of the global population has caused rapid change in Emergency Department (ED) demographics, and many elderly patients with terminal illnesses seek palliative care rather than medically intensive, high-cost care, which fails to address burdensome symptoms [1]. It is well known that the rate of ED admission increases in patients who need PC during the last months of their life, and most of the health insurance costs for care of these patients occur in the same period [1,2].

Initiation of PC for patient with end-stage disease in an ED is beneficial even though ED is not designed to care for such patients [3]. Starting PC in the emergency department offers an opportunity to support PC interventions early in the disease trajectory of patients, improves outcomes, decreases the length of stay in the hospital, results in reduced intensive care use, lowers the cost of treatment, and enhances patient and family satisfaction [4].

The EM community has placed particular emphasis on improving education in the field of PC. In 2013, ACEP (American College of Emergency Physicians) joined the "Choosing Wisely®" campaign and, in one of the recommendations, emphasized the importance of integrating PC into the ED (Emergency Department) [5].

Several studies over the past decade showed that there are educational gaps in PC training during residency. A landmark study in the United States demonstrated that only half of residents had completed any PC training in their program, and most believed that PC was a significant competence for emergency physicians [6]. At the time we designed this study, there was no structured PC or EOL educational program in the core curriculum of EM residency in Iran. As other studies suggested, there are several barriers to implementing palliative and end-of-life care curricula in EM in Iran, including short duration of country’s EM program, unavailability of experts across the country, and lack of sufficient budget for curriculum development [7].

Using the most available effective curricula for teaching PC is not practical due to their time-intensiveness and low rate of participation [8].

Using growing evidence of palliative medicine by changing physicians’ attitudes toward EOL care services is one of the critical factors in increasing the quality of services provided to end-stage disease patients in the emergency room [9]. In this study, we evaluated the attitude and its changes in EM residents before and after attending a PC workshop.

## Materials and methods

We conducted a prospective education intervention study with a standard attitude questionnaire used pre-and post-education to assess whether a short workshop could improve the residents’ attitude toward end-of-life care.

### Sampling and sample size estimation

Eligible participants included first, second, and third-year residents of the EM residency program of Tehran University of Medical Sciences of 3 teaching hospitals in 2018 without previous formal PC training who signed written consent for participation in the study.

The sample size was calculated as 40 considering data of Kitzes [10] and Levetown [11] studies using sample size estimation of comparing paired difference to achieve a power of 80% and a level of significance of 5% (two-sided), for detecting an effect size of 0.45 between pairs [12]. We used a stratified sampling considering postgraduate residency years.

### Assessment tools

Each participant reported demographic data, including age, gender, clinical/knowledge background, years of practice, previous exposure to end-stage disease in close family members, and previous consultation of the literature about EOL care in a relevant clinical situation.

In this study, the English version of the physicians’ end-of-life care attitude scale (PEAS) questionnaire was cross-culturally adapted to Persian based on recommended guidelines for translation and cross-cultural adaptation [13]. The authors also evaluated the translated version of the PEAS in a separate study. It demonstrated good reliability and validity for residents practicing EM, with a high completion rate, as shown by the absence of missing data. Internal consistency (Cronbach’s alpha coefficient) was 84%, and construct validity (Spearman coefficient for the whole questionnaire) was 0.813.

The participants were evaluated by questionnaire before the workshop and four weeks after the workshop using anonymous online software.

#### The Physicians’ End-of-Life Care Attitude Scale (PEAS)

The purpose of this questionnaire is to assess the effectiveness of palliative medicine training. This questionnaire includes 31 questions that have Likert scales with two subscales. The first is the personal communication readiness that evaluates a person’s feelings about the relationship with the end-stage patient and their family, including touching, talking about end-of-life issues, feelings and comfort when talking, and being with the patient or family. The scores range from 13–65. Second, the professional role subscale, which is an assessment of the person’s difficulties in giving the patient bad news about the disease, communicating with the patient about the future of the disease and its progress, disclosing or hiding information, discussing unexpected bad news, and feeling incomplete after the patient dies. The scores range between 18–90.

### Health professional training workshop

An independent workgroup comprised of two EM university faculty members and one palliative medicine expert-designed half-a-day accelerated PC educational program for residents. The workshop’s primary goal was to provide information that could affect participants’ attitudes toward EOL care. Additionally, we used the workshop curriculum to develop topics to be implemented during residency training. The curriculum topics are listed in Table 1. Teaching methods were chosen according to the adult learning principle, and included a didactic presentation with clinical scenarios, group discussions, and role-playing scenarios. Nine short scenarios were used to illustrate each of the main concepts. Role plays were longer and more complex and designed in a way to enable students to experience a group of 2–3 topics from many different perspectives that they may not normally encounter. At the end of the workshop, in order to ensure that all participants were exposed to all content, a small group discussion was conducted and each group was asked to complete a task and debrief among the whole group.

**Table 1:** Topics covered in the workshop.

**Recognizing a dying patient: prognostic signs at the EOL**
**Trajectory of Illness Model (recognition of PC need)**
**Approach to EOL Communication: discussing EOL care in ED**
**Goals of care: shared decision making, medical futility**
**Symptom management in the palliative patient**
**Curative treatments vs comfort care**
**Principles of a good death: patients’ fear of dying**
**Factors that originate in the health care professional**
**Multidisciplinary approach for resolving conflicts**

#### Ethical consideration

This study was approved by the ethics committee of Tehran University of Medical Sciences (IR.TUMS.MEDICINE.REC.1395.1938). All participants entered the research with personal consent, and the participants’ data remained confidential. To match pre-/post-workshop questionnaires, we asked the participant to set a five-digit identifier for both questionnaires.

### Statistical analysis

Data were entered into a Microsoft Excel spreadsheet (Microsoft Corporation, Redmond WA) and analyzed using SPSS 20.0 (SPSS Inc., Chicago, IL). All tests were two-tailed, and the level of significance was considered 0.05.

We describe quantitative data as mean and standard deviation, while qualitative data is expressed as frequency percentage. To adjust for measurement error due to regression to mean effect, we used a method developed by Roberts [14]. To compare the data, we performed paired t-tests, as well as repeated-measures ANOVA. Multiple variables were analyzed for the control of potential confounders/covariates.

## Results

This study evaluated 40 residents, 27 of whom were female (67%) and 13 male (32%). The mean age of the participants was 35.3 ± 6.5 years. There were 14 PGY1 residents, 12 PGY2 residents, and 14 PGY3 residents. Participants were not significantly different from the entire residency program according to sex, age, and year of training (P>0.05). The residents had an average of 6.2 years of clinical experience after graduation. No formal education about PC was included in the residency program of all participants. Twenty-one percent of residents with age> = 35 had previously consulted literature about EOL care vs. zero percent in the less than 35-year-old age group (p = 0.045). No association between history of End-Stage Disease in family members and previous consultation of literatures about EOL care was present (P = 0.7); there was no association between work experience and previous consultation of the literature about EOL care (p = 0.32). Table 2 provides additional demographic information.

**Table 2:** PEAS score before workshop and PEAS score difference at 4-week follow-up among group of gender, age, history of end-stage disease in close relatives, previous consultation of the literature about EOL care, years of clinical experience, and PGY (Postgraduation year).

	No. (%)	Pre-workshop score Mean ± SD	Score difference (adjusted) Mean ± SD
**Gender**			
Female	27 (67%) (67%)(67%)	88.1 ± 9.2	1.3 ± 4.8
Male	13 (32%)	84.6 ± 6.5	3.4± 6.5
**Age**			
≤ 35	21 (52%)	85.8 ± 9.2	1.9 ± 4.8
**>** 35	19 (47%)	88.2 ± 7.6	2.1 ± 6.2
**History of End-Stage Disease in family members**			
No	10 (25%)	86 ± 10.7	5.0 ± 6.0*
Yes	30 (75%)	87.3 ± 7.8	1.0 ± 4.9
**Previous consultation of the literature about EOL Care**			
No	36 (90%)	87.8 ± 8.3	2.0 ± 4.8
Yes	4 (10%)	79.2 ± 6.0	2.1 ± 10.7
**Clinical Experience (years)**			
≤ 4	20 (50%)	85.6 ± 8.3	2.7 ± 6.0
> 4	20 (50%)	88.7 ± 9.0	1.5 ± 5.0
**PGY**			
1	14 (35%)	87.6 ± 8.6	2.6 ± 5.1
2	12 (30%)	83.4 ± 9.3	1.6 ± 2.4
3	14 (35%)	89.3 ± 7.0	1.7 ± 7.6

*-P-value <0.05.

According to the PEAS questionnaire’s mean score, residents’ attitude toward EOL care at the baseline was 86.9 ± 8.5 with minimum and maximum ranges of 69 and 101, respectively. After the workshop, the average score of PEAS increased to 89 ± 6.9. The regression to the mean was applied to the baseline score of PEAS. We were comparing PEAS scores before educational intervention and four weeks after the intervention used paired-sample T-tests. There was a significant difference in scores (mean difference = 2.05 ± 5.4, P = 0.023).

No significant difference in baseline PEAS score was present between groups of gender, age, PGY, clinical experience history, and history of dealing with a close relative with the end-stage disease (Table 2). In comparing pre-workshop and post-workshop scores, the only group with a statistically significant improvement was the group with no history of end-stage disease in their families (p = 0.045, Table 2).

Baseline personal communication readiness subscale and professional role subscale scores were 36.7±5.6 and 50.2±4.1, respectively. The adjusted increase in the score at 4-week follow-up was 1.15±3.8 and 0.9±2.9 in first and second subscales, respectively (effect size 0.30 and 0.310), which only the second subscale increase was statistically significant (p = 0.030).

## Discussion

Caring for patients with end-stage disease for EM physicians is considered a regular part of their daily practice. In an attempt to integrate PC training into the EM residency program, we designed the study to explore whether a condensed educational program would cause a lasting impression on the attitude of EM residences towards palliative care.

Overall, the results of this study indicate that a short-term educational intervention focused on the main topics of EOL care designed by an expert team can have a significant effect on promoting residents’ attitudes toward EOL care.

To control for the effect of possible demographics and residents’ past medical and/or personal experience, we evaluated several variables with a possible association with their baseline attitude and the magnitude of change toward EOL care. We also separated the research workgroup and education workgroup to minimize bias in designing educational materials.

We compared the baseline PEAS score, which reflects attitude toward EOL care, based on demographics or clinical knowledge/background. One may expect a higher baseline PEAS score in residents with higher clinical experience or previous exposure to end-stage disease in their families or those who had self-studied about PC. However, there was no statistical difference between these groups. Interestingly older residents had a significantly higher rate of previous consultation of the literatures about EOL care.

The only background variable that was predictive of improvement in attitude in a 4-week follow-up was the absence of encounters with the end-stage disease in residents’ close family members. This shows that although exposure to terminally ill patients frequently happens during the doctors’ career, lack of proper knowledge and training may contribute to anxiety and negative attitude toward the dying patient. However, with a close dying relative, exposure to intense emotions can be a learning opportunity for the doctor, and attitude change may forms through an adaptive mechanism [15].

Several studies demonstrated the lack of sufficient PC training in the EM residency curriculum and reported PC is a significant competency for EM physicians [6,7,16,17]. Despite recognizing the value of PC education, there is no consensus of optimal volume or method for training. Goldonowicz et al., in 2018, described that simulation was an effective tool for learning PC [18]. Wright et al. in 2019 used an ED-based PC communication skills training workshop and showed it is beneficial for residents’ approach to patients and instructing PC skills to learners [19]. An Australian study by Jelinek et al showed that even brief exposure of a single day appears to change ED clinicians’ attitude and practice in PC [20]. In DeVader et al. study, 4 hours of lecturing about PC for EM residents was sufficient to develop a long-lasting knowledge and improved attitude and practice of PC [21]. Nam et al. reported experience of hospice education as a predictor of attitude to EOL care [22]. According to questionnaire scores of our participants with previous consultation of the literature it appears that self-directed learning had no effect on residents’ attitude toward EOL care. Putting all findings together, the importance of incorporating at least a condensed PC training course into residents’ curricula is a necessity.

Our study showed an improvement in participants’ attitudes at the four-week follow-up compared to baseline. However, this study has potential limitations. This is a single-center study with limited sample size and short term follow-up that may not apply to other countries. There is no predefined significant threshold for improved attitude nor our study measured change in behavior after graduation. Further research must focus on the fact that the best method of introducing PC into EM training program is still not clear, and lack of availability of proper assessment tools for measuring knowledge, attitude and practice of physicians about EOL care and defining the desired threshold of each parts to address the needs of EM physicians.

## Conclusions

Our study suggests that a multidisciplinary designed short course workshop can change and improve the attitudes of EM residents towards EOL care in the ED. The optimal design and characteristics of this educational intervention remain to be defined by further studies. Still, there is a need to incorporate PC training into residents’ curricula with PC specialists and/or coordination between residency programs.

## Supporting information

S1 Dataset (PDF)

S1 Questionaire (PDF)
