# Supplementary material for: Attitude toward end-of-life care in emergency medicine residents- can a short workshop make a difference?
Source: PLoS One. 2023 Jan 11;18(1):e0280229. doi: 10.1371/journal.pone.0280229 (PMC9833511; doi:10.1371/journal.pone.0280229)
Supplement: S1 Dataset — (PDF) [file pone.0280229.s001.pdf]

```
DATASET ACTIVATE DataSet1.
DATASET CLOSE DataSet0.

SAVE OUTFILE='C:\Users\user\Desktop\Analysis Payanname\pre-post analysis.sav'
  /COMPRESSED.
USE ALL.
COMPUTE filter_$=( ~ MISSING(q1p)).
VARIABLE LABELS filter_$ ' ~ MISSING(q1p) (FILTER)'.
VALUE LABELS filter_$ 0 'Not Selected' 1 'Selected'.
FORMATS filter_$ (f1.0).
FILTER BY filter_$.
EXECUTE
FREQUENCIES VARIABLES=Gender Age PGY Clin_exp
  /NTILES=4
  /STATISTICS=STDDEV MINIMUM MAXIMUM MEAN
  /ORDER=ANALYSIS



FILTER OFF.
USE ALL.
EXECUTE
COMPUTE PEAS_pre=SUM(q1,q2,q3,q4,q5,q6,q7,q8,q9,q10,q11,q12,q13,q14,q15,q16,q17,q18,q19,q20,q2
1,q22,
    q23,q24,q25,q26,q27,q28,q29,q30,q31).
EXECUTE
COMPUTE PEAS_post=SUM(q1p,q2p,q3p,q4p,q5p,q6p,q7p,q8p,q9p,q10p,q11p,q12p,q13p,q14p,q15p,q16p,
q17p,
    q18p,q19p,q20p,q21p,q22p,q23p,q24p,q25p,q26p,q27p,q28p,q29p,q30p,q31p).
EXECUTE
COMPUTE PEAS_diff=PEAS_post - PEAS_pre.
EXECUTE
SUMMARIZE
  /TABLES=Name PEAS_diff
  /FORMAT=VALIDLIST NOCASENUM TOTAL LIMIT=100
  /TITLE='Case Summaries'
  /MISSING=VARIABLE
  /CELLS=COUNT.



SORT CASES BY PGY (A).
USE ALL.
COMPUTE filter_$=( ~ MISSING(q1p)).
VARIABLE LABELS filter_$ ' ~ MISSING(q1p) (FILTER)'.
VALUE LABELS filter_$ 0 'Not Selected' 1 'Selected'.
```

```
FORMATS filter_$ (f1.0).
FILTER BY filter_$.
EXECUTE.
```

```
FREQUENCIES VARIABLES=Gender Age PGY Clin_exp exposure study
/NTILES=4
/STATISTICS=STDDEV MEAN MEDIAN
/HISTOGRAM NORMAL
/ORDER=ANALYSIS
```

Frequencies

Statistics

		Gender	( )				
N	Valid	40	40	40	38	40	40
	Missing	0	0	0	2	0	0
Mean		1.33	35.3250	2.00	6.2605	1.75	1.10
Median		1.00	34.5000	2.00	4.0000	2.00	1.00
Std. Deviation		.474	6.50596	.847	6.10049	.439	.304
Percentiles	25	1.00	30.0000	1.00	1.3750	1.25	1.00
	50	1.00	34.5000	2.00	4.0000	2.00	1.00
	75	2.00	39.0000	3.00	10.0000	2.00	1.00

Frequency Table

Gender

		Frequency	Percent	Valid Percent	Cumulative Percent
Valid	Female	27	67.5	67.5	67.5
	Male	13	32.5	32.5	100.0
	Total	40	100.0	100.0	

		Frequency	Percent	Valid Percent	Cumulative Percent
Valid	1	14	35.0	35.0	35.0
	2	12	30.0	30.0	65.0
	3	14	35.0	35.0	100.0
	Total	40	100.0	100.0	

		Frequency	Percent	Valid Percent	Cumulative Percent
Valid	No	10	25.0	25.0	25.0
	Yes	30	75.0	75.0	100.0
	Total	40	100.0	100.0	

		Frequency	Percent	Valid Percent	Cumulative Percent
Valid	No	36	90.0	90.0	90.0
	Yes	4	10.0	10.0	100.0
	Total	40	100.0	100.0	

Histogram

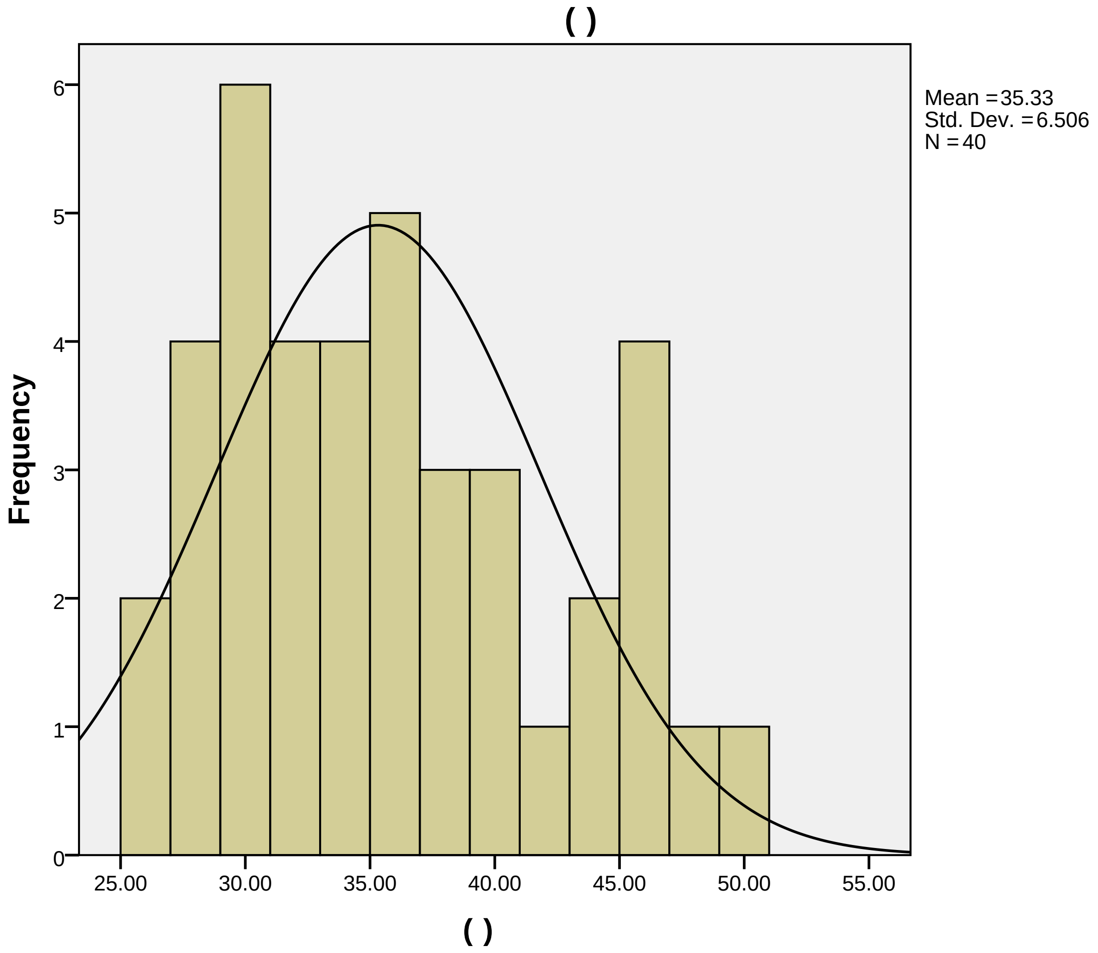
( )
Mean = 35.33
Std. Dev. = 6.506
N = 40
Frequency
0
1
2
3
4
5
6
25.00
30.00
35.00
40.00
45.00
50.00
55.00
( )

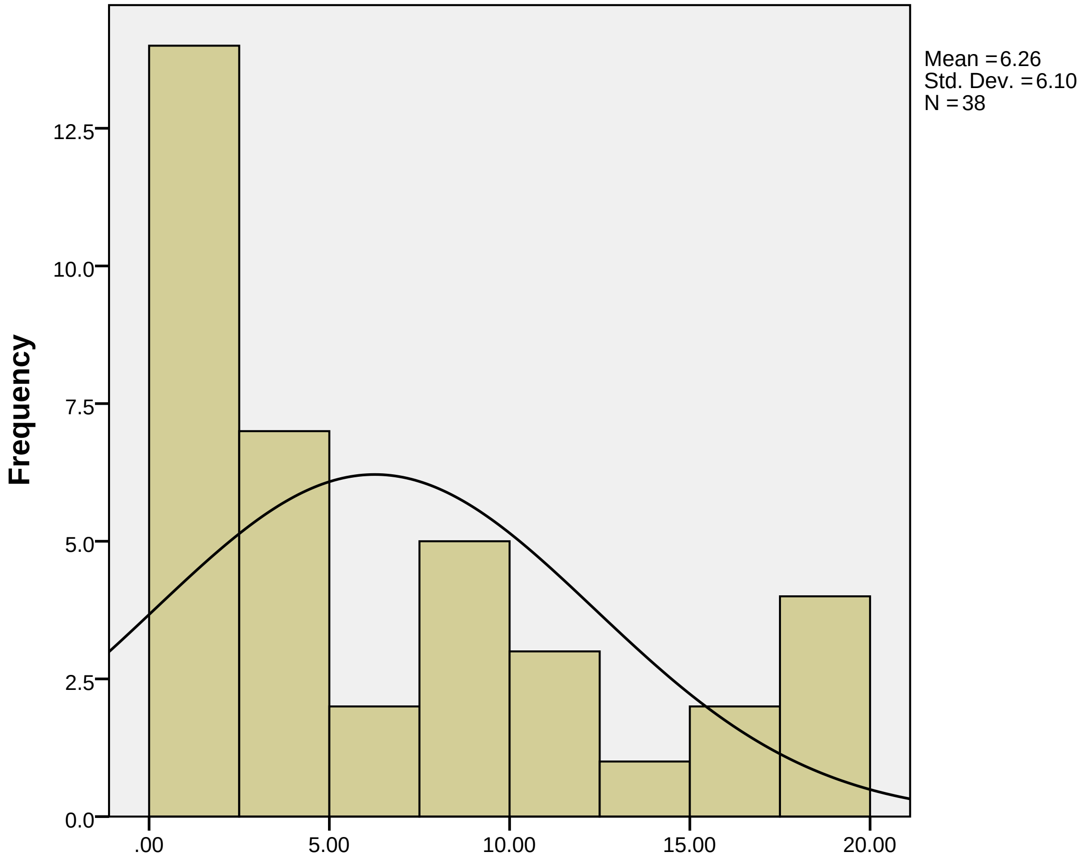


```
FREQUENCIES VARIABLES=Gender Age PGY Clin_exp exposure study
/NTILES=4
/STATISTICS=STDDEV MEAN MEDIAN
/PIECHART PERCENT
/ORDER=ANALYSIS.
```

Frequencies

Pie Chart

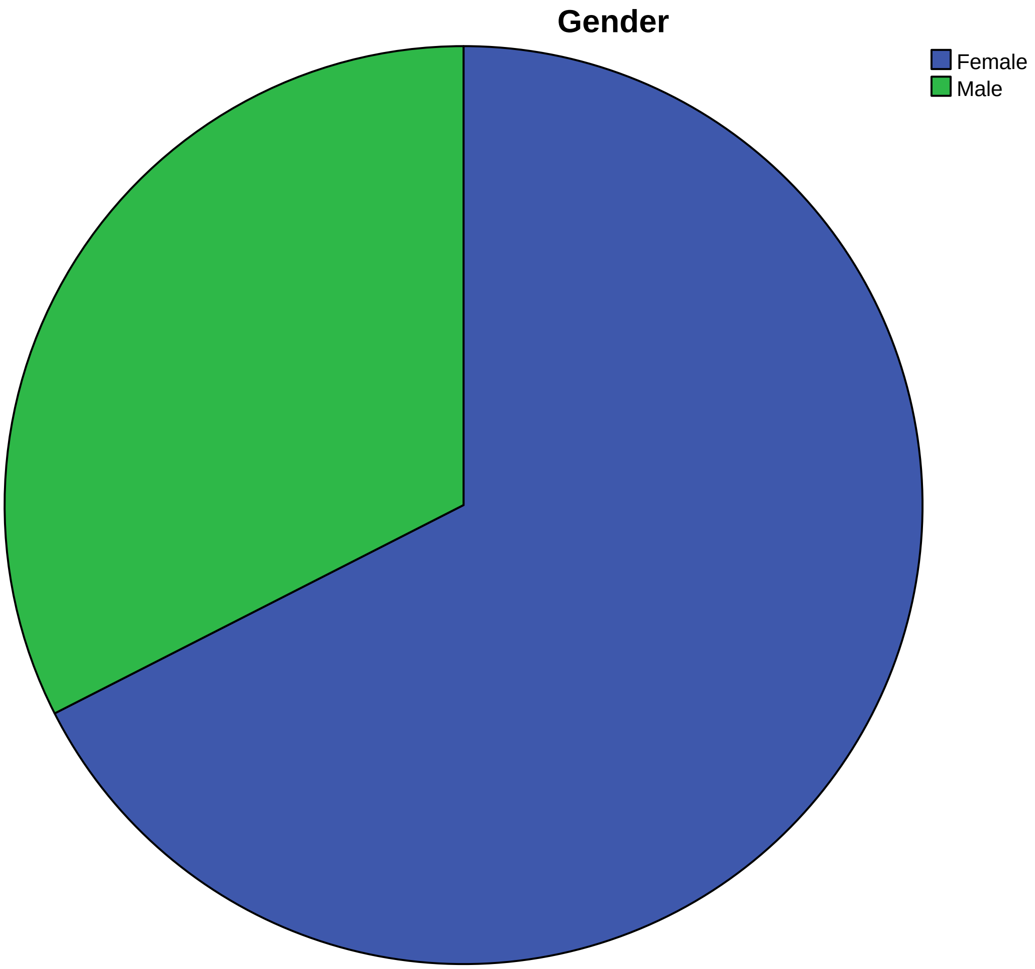
Gender
Female
Male

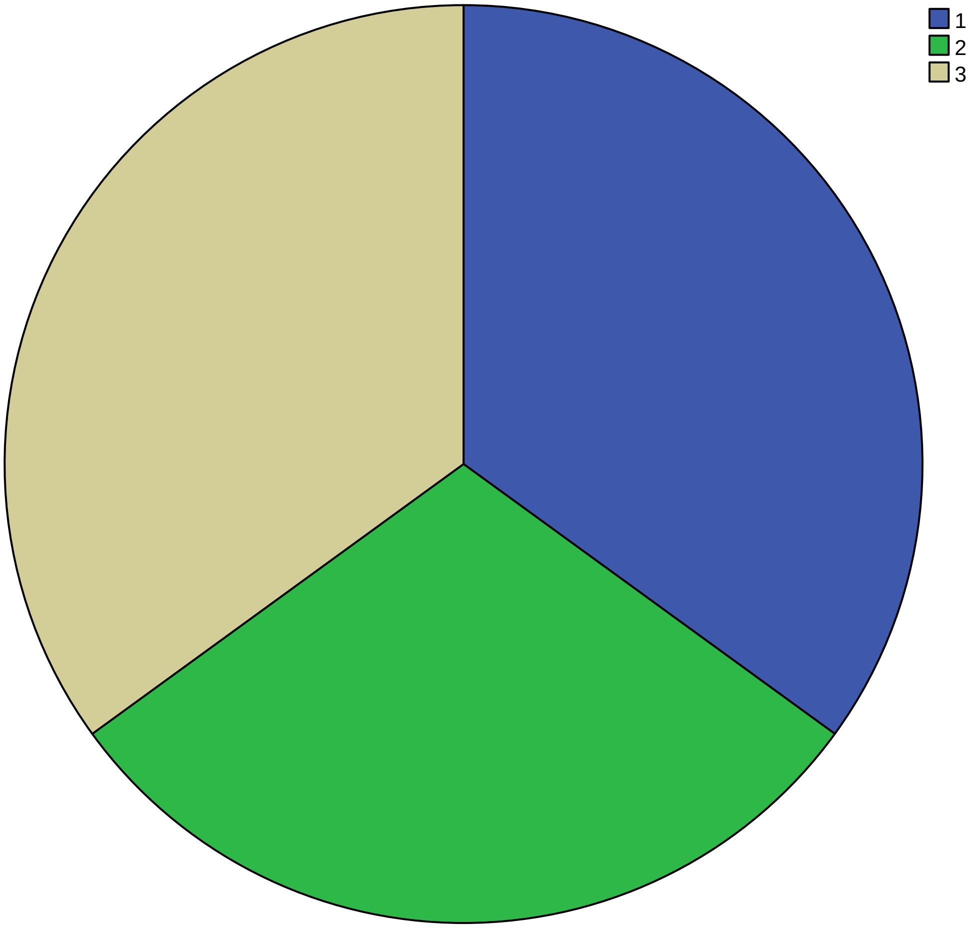
1
2
3

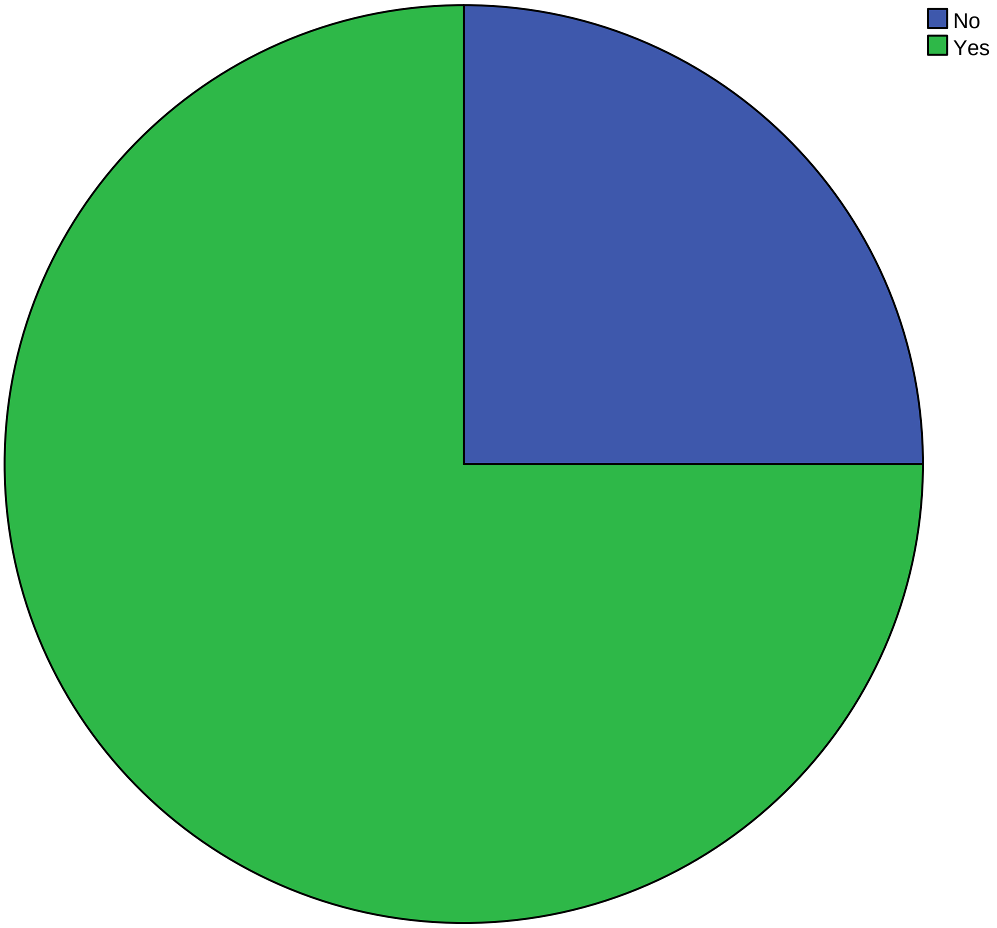
No
Yes

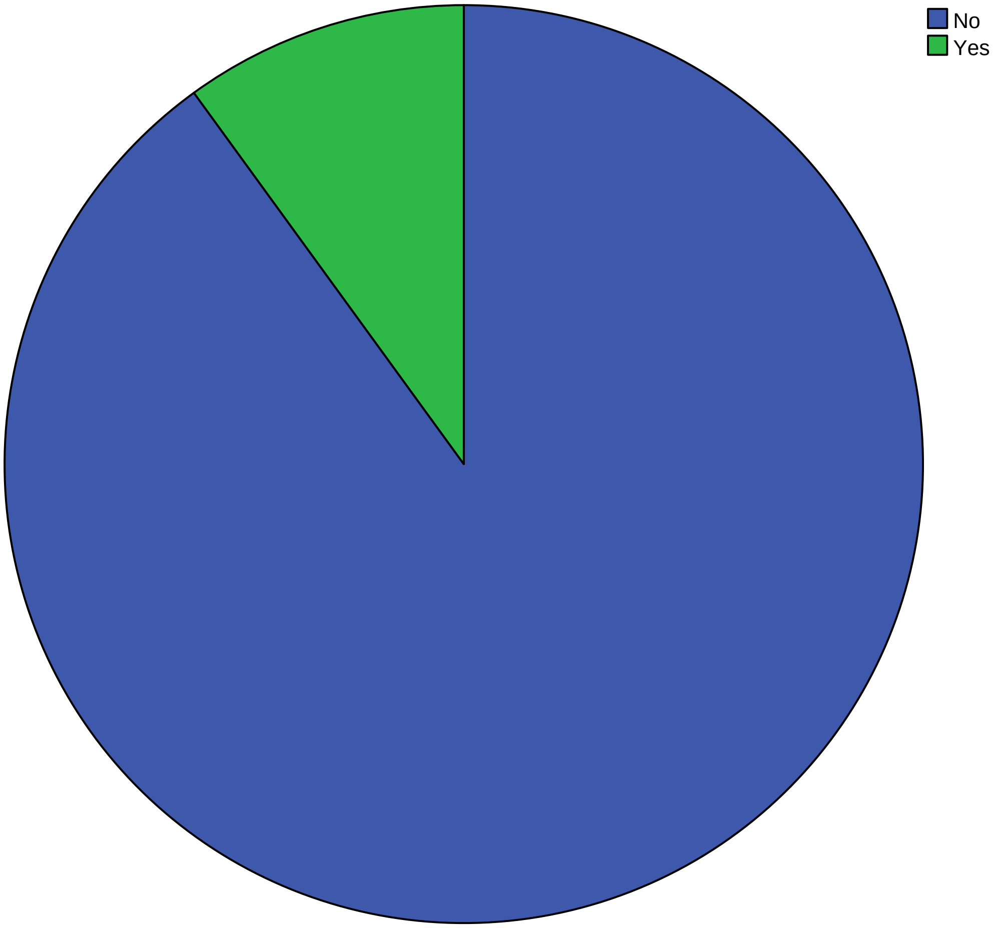


```
  RECODE Age (0 thru 35=1) (36 thru 100=2) INTO age_g.
VARIABLE LABELS age_g 'Group Age'.
EXECUTE.
SORT CASES BY Age (A).
RECODE Clin_exp (0 thru 4=1) (4.1 thru Highest=2) INTO Clin_exp_grp.
EXECUTE.
FREQUENCIES VARIABLES=age_g Clin_exp_grp
  /PIECHART PERCENT
  /ORDER=ANALYSIS.
```

Frequency Table

		Frequency	Percent	Valid Percent	Cumulative Percent
Valid	=<35	21	52.5	52.5	52.5
	>35	19	47.5	47.5	100.0
	Total	40	100.0	100.0	

		Frequency	Percent	Valid Percent	Cumulative Percent
Valid	=<4	20	50.0	52.6	52.6
	>4	18	45.0	47.4	100.0
	Total	38	95.0	100.0	
Missing	System	2	5.0		
Total		40	100.0		

Pie Chart

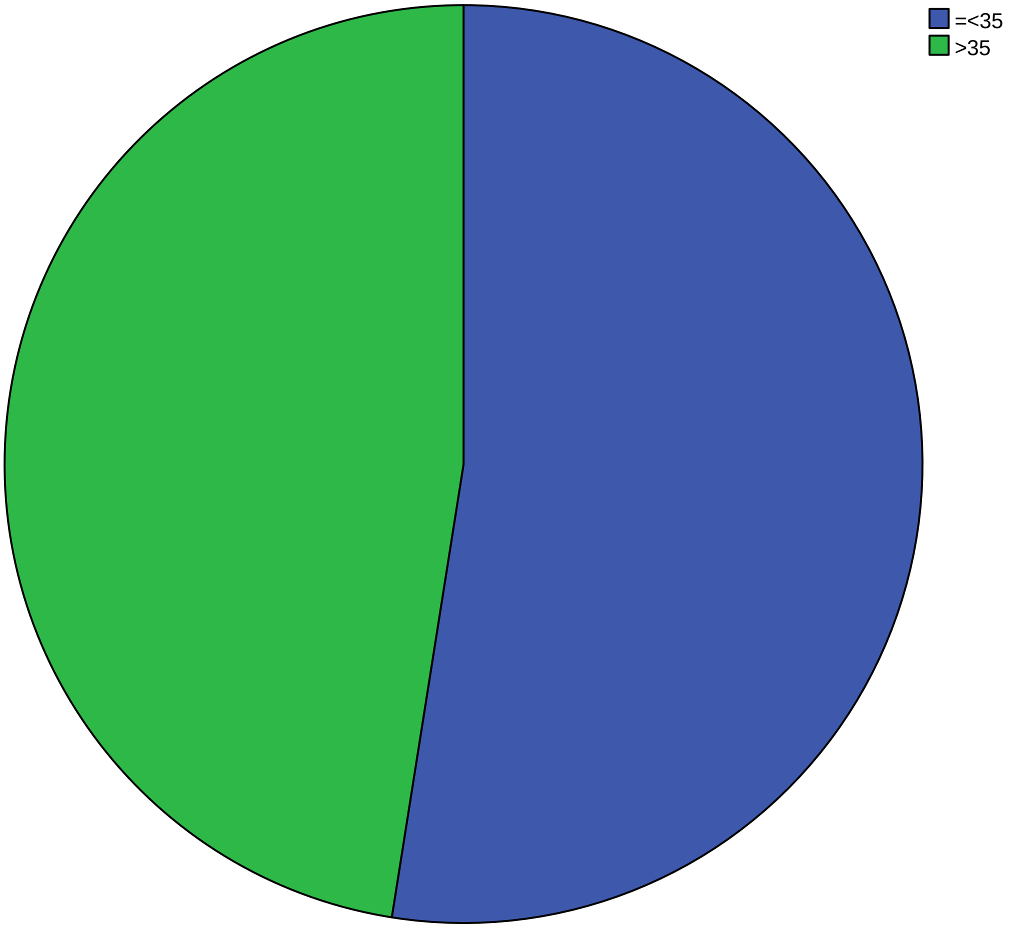
=<35
>35

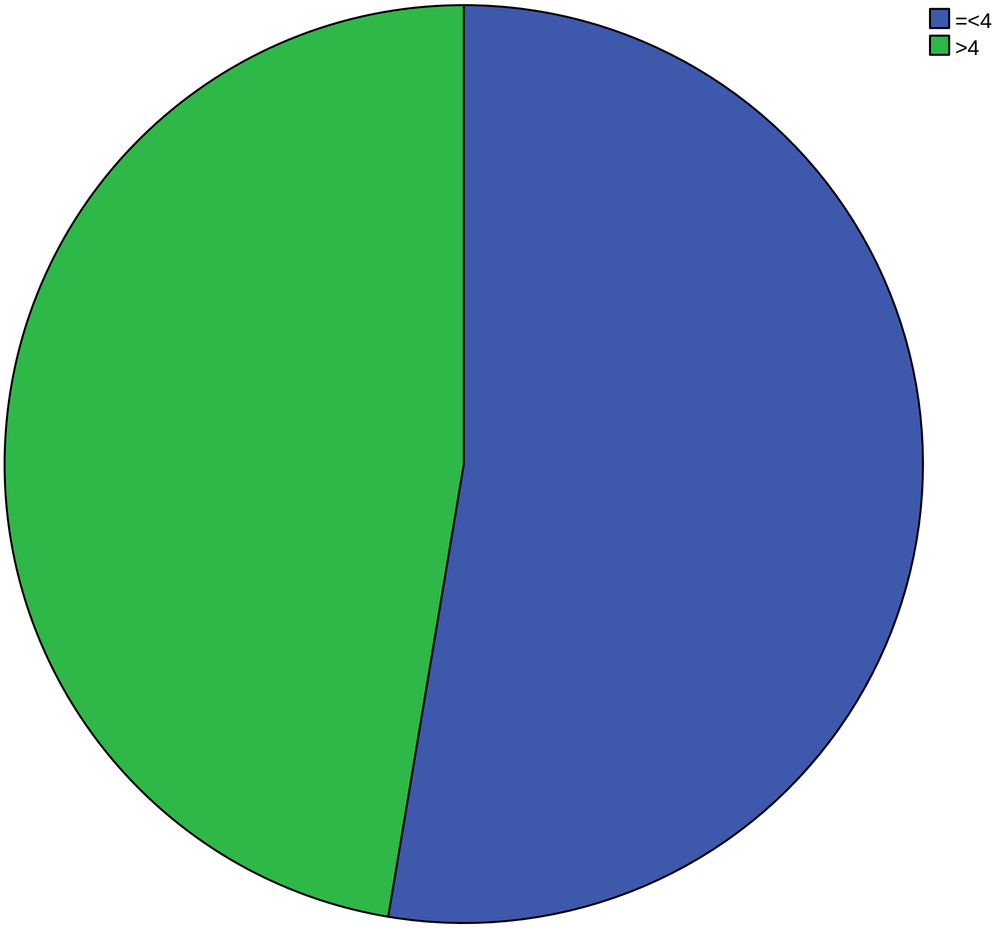


```
FREQUENCIES VARIABLES=PEAS_pre
/NTILES=4
/STATISTICS=STDDEV MINIMUM MAXIMUM MEAN MEDIAN MODE
/HISTOGRAM NORMAL
/ORDER=ANALYSIS.
```

Frequencies

Statistics

PEAS score baseline

N	Valid	40
Mean		86.9750
Median		86.5000
Mode		85.00
Std. Deviation		8.53195
Minimum		69.00
Maximum		101.00
Percentiles	25	82.2500
	50	86.5000
	75	93.7500

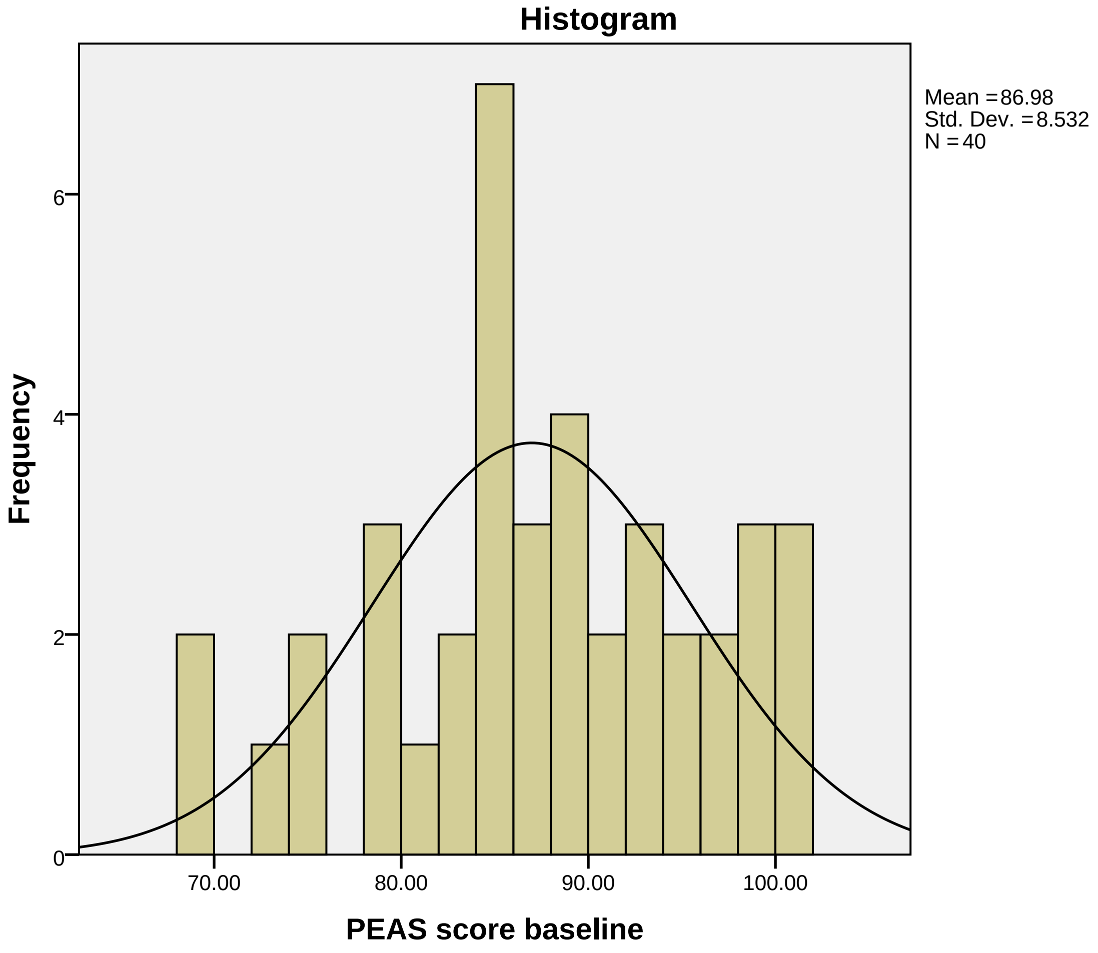


```
T-TEST PAIRS=PEAS_pre WITH PEAS_post (PAIRED)
/CRITERIA=CI(.9500)
```

/MISSING=ANALYSIS

T-Test

Paired Samples Statistics

		Mean	N	Std. Deviation	Std. Error Mean
Pair 1	PEAS score baseline	86.9750	40	8.53195	1.34902
	PEAS score after workshop	89.0250	40	6.93740	1.09690

Paired Samples Correlations

		N	Correlation	Sig.
Pair 1	PEAS score baseline & PEAS score after workshop	40	.631	.000

Paired Samples Test_Before adjustment

		Paired Differences					t
		Mean	Std. Deviation	Std. Error Mean	95% Confidence Interval of the Difference		
					Lower	Upper	
Pair 1	PEAS score baseline - PEAS score after workshop	-2.05000	6.79725	1.07474	-4.22386	.12386	-1.907

Paired Samples Test_Before adjustment

		df	Sig. (2-tailed)
Pair 1	PEAS score baseline - PEAS score after workshop	39	.064

DATASET ACTIVATE DataSet1.

```
SAVE OUTFILE='C:\Users\user\Desktop\Analysis Payanname\pre-post analysis.sav'
  /COMPRESSED.
T-TEST PAIRS=q1 q2 q3 q4 q5 q6 q7 q8 q9 q10 q11 q12 q13 q14 q15 q16 q17 q18 q19 q20 q21 q22 q23 q
24
    q25 q26 q27 q28 q29 q30 q31 WITH q1p q2p q3p q4p q5p q6p q7p q8p q9p q10p q11p q12p q13p q14p q
15p
    q16p q17p q18p q19p q20p q21p q22p q23p q24p q25p q26p q27p q28p q29p q30p q31p (PAIRED)
  /CRITERIA=CI(.9500)
  /MISSING=ANALYSIS
```

T-Test

Paired Samples Statistics

		Mean	N	Std. Deviation	Std. Error Mean
Pair 1	q1	2.63	40	1.170	.185
	q1p	2.70	40	1.305	.206
Pair 2	q2	1.78	40	.620	.098
	q2p	1.75	40	.543	.086
Pair 3	q3	3.55	40	.876	.138
	q3p	3.68	40	.797	.126
Pair 4	q4	3.38	40	.897	.142
	q4p	3.73	40	.816	.129
Pair 5	q5	3.03	40	1.187	.188
	q5p	3.20	40	1.137	.180
Pair 6	q6	2.90	40	1.128	.178
	q6p	2.73	40	1.037	.164
Pair 7	q7	2.60	40	1.128	.178
	q7p	3.08	40	1.023	.162
Pair 8	q8	3.00	40	1.086	.172
	q8p	3.43	40	.903	.143
Pair 9	q9	2.17	40	.903	.143
	q9p	2.63	40	.952	.151
Pair 10	q10	3.05	40	1.085	.172
	q10p	2.80	40	.992	.157
Pair 11	q11	2.55	40	1.300	.206
	q11p	2.48	40	1.037	.164
Pair 12	q12	2.95	40	1.176	.186
	q12p	2.63	40	1.079	.171
Pair 13	q13	3.13	40	1.042	.165
	q13p	3.05	40	1.108	.175
Pair 14	q14	3.18	40	.931	.147
	q14p	3.25	40	.840	.133

Paired Samples Statistics

		Mean	N	Std. Deviation	Std. Error Mean
Pair 15	q15	2.95	40	1.011	.160
	q15p	3.08	40	.888	.140
Pair 16	q16	2.25	40	.981	.155
	q16p	2.38	40	.952	.151
Pair 17	q17	3.98	40	.920	.145
	q17p	4.22	40	.698	.110
Pair 18	q18	3.70	40	.966	.153
	q18p	3.90	40	.672	.106
Pair 19	q19	3.10	40	1.150	.182
	q19p	3.35	40	1.099	.174
Pair 20	q20	1.33	40	.474	.075
	q20p	1.45	40	.504	.080
Pair 21	q21	3.00	40	1.013	.160
	q21p	2.98	40	.947	.150
Pair 22	q22	2.75	40	1.149	.182
	q22p	2.20	40	.791	.125
Pair 23	q23	2.25	40	1.296	.205
	q23p	2.45	40	1.011	.160
Pair 24	q24	2.90	40	1.172	.185
	q24p	2.40	40	.778	.123
Pair 25	q25	2.13	40	.853	.135
	q25p	2.10	40	.709	.112
Pair 26	q26	2.65	40	1.027	.162
	q26p	2.68	40	1.023	.162
Pair 27	q27	3.50	40	.877	.139
	q27p	3.28	40	.905	.143
Pair 28	q28	2.73	40	1.198	.189
	q28p	2.68	40	.997	.158
Pair 29	q29	3.35	40	1.001	.158
	q29p	3.78	40	.920	.145
Pair 30	q30	2.10	40	.900	.142
	q30p	2.48	40	.987	.156
Pair 31	q31	2.45	40	.932	.147
	q31p	2.55	40	.876	.138

Paired Samples Correlations

		N	Correlation	Sig.
Pair 1	q1 & q1p	40	.529	.000
Pair 2	q2 & q2p	40	.210	.194
Pair 3	q3 & q3p	40	.410	.009
Pair 4	q4 & q4p	40	.670	.000
Pair 5	q5 & q5p	40	.433	.005
Pair 6	q6 & q6p	40	.546	.000
Pair 7	q7 & q7p	40	.516	.001
Pair 8	q8 & q8p	40	.392	.012
Pair 9	q9 & q9p	40	.585	.000
Pair 10	q10 & q10p	40	.343	.030
Pair 11	q11 & q11p	40	.448	.004
Pair 12	q12 & q12p	40	.248	.123
Pair 13	q13 & q13p	40	.572	.000
Pair 14	q14 & q14p	40	.500	.001
Pair 15	q15 & q15p	40	.461	.003
Pair 16	q16 & q16p	40	.199	.218
Pair 17	q17 & q17p	40	.769	.000
Pair 18	q18 & q18p	40	.506	.001
Pair 19	q19 & q19p	40	.296	.064
Pair 20	q20 & q20p	40	.660	.000
Pair 21	q21 & q21p	40	.588	.000
Pair 22	q22 & q22p	40	.395	.012
Pair 23	q23 & q23p	40	.421	.007
Pair 24	q24 & q24p	40	.607	.000
Pair 25	q25 & q25p	40	.572	.000
Pair 26	q26 & q26p	40	.426	.006
Pair 27	q27 & q27p	40	.726	.000
Pair 28	q28 & q28p	40	.202	.211
Pair 29	q29 & q29p	40	.478	.002
Pair 30	q30 & q30p	40	.609	.000
Pair 31	q31 & q31p	40	.537	.000

Paired Samples Test

		Paired Differences					t	df
		Mean	Std. Deviation	Std. Error Mean	95% Confidence Interval of the Difference			
					Lower	Upper		
Pair 1	q1 - q1p	-.075	1.207	.191	-.461	.311	-.393	39
Pair 2	q2 - q2p	.025	.733	.116	-.210	.260	.216	39
Pair 3	q3 - q3p	-.125	.911	.144	-.416	.166	-.868	39
Pair 4	q4 - q4p	-.350	.700	.111	-.574	-.126	-3.163	39
Pair 5	q5 - q5p	-.175	1.238	.196	-.571	.221	-.894	39
Pair 6	q6 - q6p	.175	1.035	.164	-.156	.506	1.069	39
Pair 7	q7 - q7p	-.475	1.062	.168	-.815	-.135	-2.829	39
Pair 8	q8 - q8p	-.425	1.107	.175	-.779	-.071	-2.429	39
Pair 9	q9 - q9p	-.450	.846	.134	-.721	-.179	-3.365	39
Pair 10	q10 - q10p	.250	1.193	.189	-.132	.632	1.325	39
Pair 11	q11 - q11p	.075	1.248	.197	-.324	.474	.380	39
Pair 12	q12 - q12p	.325	1.385	.219	-.118	.768	1.484	39
Pair 13	q13 - q13p	.075	.997	.158	-.244	.394	.476	39
Pair 14	q14 - q14p	-.075	.888	.140	-.359	.209	-.534	39
Pair 15	q15 - q15p	-.125	.992	.157	-.442	.192	-.797	39
Pair 16	q16 - q16p	-.125	1.223	.193	-.516	.266	-.646	39
Pair 17	q17 - q17p	-.250	.588	.093	-.438	-.062	-2.687	39
Pair 18	q18 - q18p	-.200	.853	.135	-.473	.073	-1.482	39
Pair 19	q19 - q19p	-.250	1.335	.211	-.677	.177	-1.184	39
Pair 20	q20 - q20p	-.125	.404	.064	-.254	.004	-1.955	39
Pair 21	q21 - q21p	.025	.891	.141	-.260	.310	.177	39
Pair 22	q22 - q22p	.550	1.108	.175	.196	.904	3.139	39
Pair 23	q23 - q23p	-.200	1.265	.200	-.605	.205	-1.000	39
Pair 24	q24 - q24p	.500	.934	.148	.201	.799	3.387	39
Pair 25	q25 - q25p	.025	.733	.116	-.210	.260	.216	39
Pair 26	q26 - q26p	-.025	1.097	.174	-.376	.326	-.144	39
Pair 27	q27 - q27p	.225	.660	.104	.014	.436	2.157	39
Pair 28	q28 - q28p	.050	1.395	.221	-.396	.496	.227	39
Pair 29	q29 - q29p	-.425	.984	.156	-.740	-.110	-2.731	39
Pair 30	q30 - q30p	-.375	.838	.132	-.643	-.107	-2.831	39
Pair 31	q31 - q31p	-.100	.871	.138	-.379	.179	-.726	39

Paired Samples Test

		Sig. (2-tailed)
Pair 1	q1 - q1p	.696
Pair 2	q2 - q2p	.830
Pair 3	q3 - q3p	.391
Pair 4	q4 - q4p	.003
Pair 5	q5 - q5p	.377
Pair 6	q6 - q6p	.291
Pair 7	q7 - q7p	.007
Pair 8	q8 - q8p	.020
Pair 9	q9 - q9p	.002
Pair 10	q10 - q10p	.193
Pair 11	q11 - q11p	.706
Pair 12	q12 - q12p	.146
Pair 13	q13 - q13p	.637
Pair 14	q14 - q14p	.596
Pair 15	q15 - q15p	.430
Pair 16	q16 - q16p	.522
Pair 17	q17 - q17p	.011
Pair 18	q18 - q18p	.146
Pair 19	q19 - q19p	.243
Pair 20	q20 - q20p	.058
Pair 21	q21 - q21p	.860
Pair 22	q22 - q22p	.003
Pair 23	q23 - q23p	.323
Pair 24	q24 - q24p	.002
Pair 25	q25 - q25p	.830
Pair 26	q26 - q26p	.886
Pair 27	q27 - q27p	.037
Pair 28	q28 - q28p	.822
Pair 29	q29 - q29p	.009
Pair 30	q30 - q30p	.007
Pair 31	q31 - q31p	.472

```
GLM PEAS_pre PEAS_post BY age_g
/WSFACTOR=peasscore 2 Polynomial
```

```
/METHOD=SSTYPE(3)
/CRITERIA=ALPHA(.05)
/WSDESIGN=peasscore
/DESIGN=age_g.
```

General Linear Model

Within-Subjects Factors

Measure: MEASURE_1

peasscore	Dependent Variable
1	PEAS_pre
2	PEAS_post

Between-Subjects Factors

	Value Label	N
1.00	=<35	21
2.00	>35	19

Multivariate Tests[a]

Effect		Value	F	Hypothesis df	Error df	Sig.
peasscore	Pillai's Trace	.084	3.486[b]	1.000	38.000	.070
	Wilks' Lambda	.916	3.486[b]	1.000	38.000	.070
	Hotelling's Trace	.092	3.486[b]	1.000	38.000	.070
	Roy's Largest Root	.092	3.486[b]	1.000	38.000	.070
peasscore * age_g	Pillai's Trace	.003	.102[b]	1.000	38.000	.751
	Wilks' Lambda	.997	.102[b]	1.000	38.000	.751
	Hotelling's Trace	.003	.102[b]	1.000	38.000	.751
	Roy's Largest Root	.003	.102[b]	1.000	38.000	.751

a. Design: Intercept + age_g
Within Subjects Design: peasscore

b. Exact statistic

Mauchly's Test of Sphericity[a]

Measure: MEASURE_1

Within Subjects Effect	Mauchly's W	Approx. Chi-Square	df	Sig.	Epsilon[b]		
					Greenhouse-Geisser	Huynh-Feldt	Lower-bound
peasscore	1.000	.000	0	.	1.000	1.000	1.000

Tests the null hypothesis that the error covariance matrix of the orthonormalized transformed dependent variables is proportional to an identity matrix.

a. Design: Intercept + age_g
Within Subjects Design: peasscore

b. May be used to adjust the degrees of freedom for the averaged tests of significance. Corrected tests are displayed in the Tests of Within-Subjects Effects table.

Tests of Within-Subjects Effects

Measure: MEASURE_1

Source		Type III Sum of Squares	df	Mean Square	F	Sig.
peasscore	Sphericity Assumed	82.421	1	82.421	3.486	.070
	Greenhouse-Geisser	82.421	1.000	82.421	3.486	.070
	Huynh-Feldt	82.421	1.000	82.421	3.486	.070
	Lower-bound	82.421	1.000	82.421	3.486	.070
peasscore * age_g	Sphericity Assumed	2.421	1	2.421	.102	.751
	Greenhouse-Geisser	2.421	1.000	2.421	.102	.751
	Huynh-Feldt	2.421	1.000	2.421	.102	.751
	Lower-bound	2.421	1.000	2.421	.102	.751
Error(peasscore)	Sphericity Assumed	898.529	38	23.645		
	Greenhouse-Geisser	898.529	38.000	23.645		
	Huynh-Feldt	898.529	38.000	23.645		
	Lower-bound	898.529	38.000	23.645		

Tests of Within-Subjects Contrasts

Measure: MEASURE_1

Source	peasscore	Type III Sum of Squares	df	Mean Square	F	Sig.
peasscore	Linear	82.421	1	82.421	3.486	.070
peasscore * age_g	Linear	2.421	1	2.421	.102	.751
Error(peasscore)	Linear	898.529	38	23.645		

Tests of Between-Subjects Effects

Measure: MEASURE_1

Transformed Variable: Average

Source	Type III Sum of Squares	df	Mean Square	F	Sig.
Intercept	618675.4	1	618675.4	6294.760	.000
age_g	80.201	1	80.201	.816	.372
Error	3734.799	38	98.284		

```
GLM PEAS_pre PEAS_post BY age_g
/WSFACTOR=peasscore 2 Polynomial
/METHOD=SSTYPE(3)
/PLOT=PROFILE(age_g*peasscore)
/CRITERIA=ALPHA(.05)
/WSDESIGN=peasscore
/DESIGN=age_g.
```

General Linear Model

Profile Plots

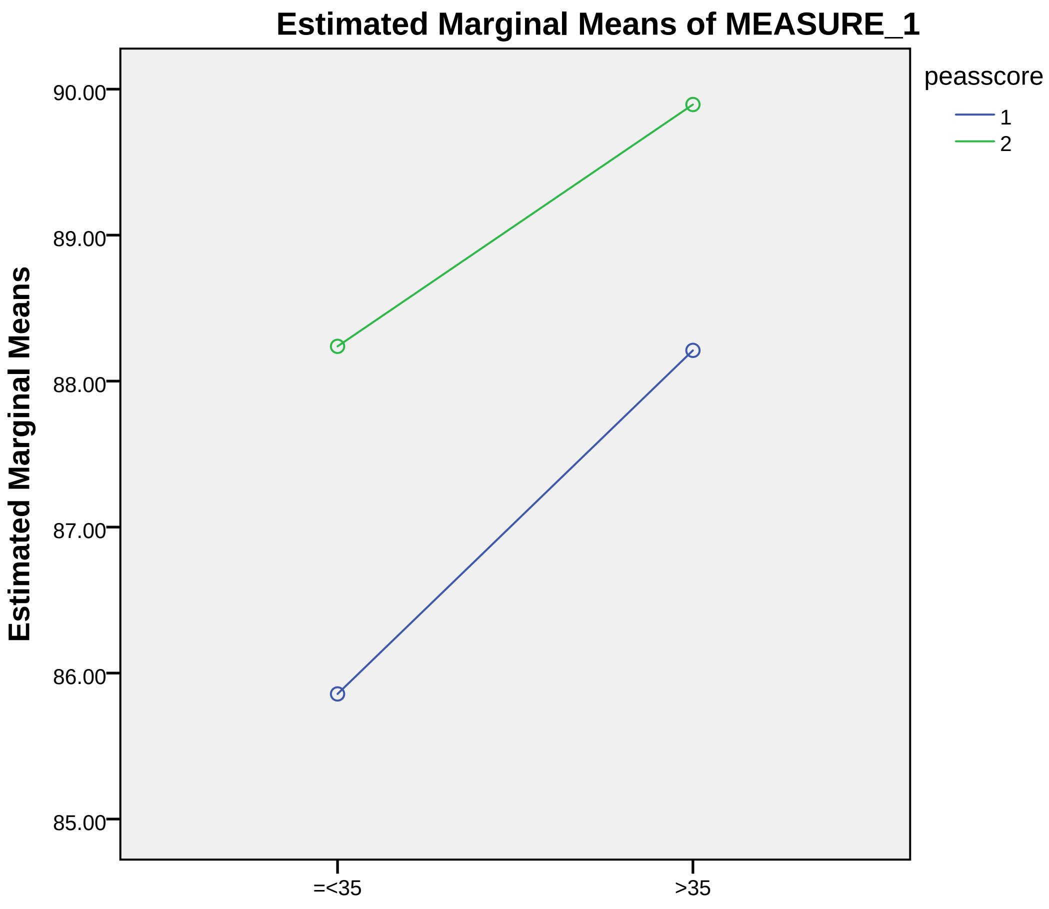


```
GLM PEAS_pre PEAS_post BY age_g
/WSFACTOR=peasscore 2 Polynomial
/METHOD=SSTYPE(3)
/PLOT=PROFILE(peasscore*age_g)
/CRITERIA=ALPHA(.05)
/WSDESIGN=peasscore
/DESIGN=age_g.
```

```
GLM PEAS_pre PEAS_post BY exposure PGY study
/WSFACTOR=peasscore 2 Polynomial
/METHOD=SSTYPE(3)
/PLOT=PROFILE(peasscore*exposure peasscore*PGY peasscore*study)
/CRITERIA=ALPHA(.05)
/WSDESIGN=peasscore
/DESIGN=exposure PGY study exposure*PGY exposure*study PGY*study exposure*PGY*study.
```

General Linear Model

Within-Subjects Factors

Measure: MEASURE_1

peasscore	Dependent Variable
1	PEAS_pre
2	PEAS_post

Between-Subjects Factors

	Value Label	N
1	No	10
2	Yes	30
1		14
2		12
3		14
1	No	36
2	Yes	4

Multivariate Tests[a]

Effect		Value	F	Hypothesis df	Error df	Sig.
peasscore	Pillai's Trace	.252	10.461[b]	1.000	31.000	.003
	Wilks' Lambda	.748	10.461[b]	1.000	31.000	.003
	Hotelling's Trace	.337	10.461[b]	1.000	31.000	.003
	Roy's Largest Root	.337	10.461[b]	1.000	31.000	.003
peasscore * exposure	Pillai's Trace	.267	11.287[b]	1.000	31.000	.002
	Wilks' Lambda	.733	11.287[b]	1.000	31.000	.002
	Hotelling's Trace	.364	11.287[b]	1.000	31.000	.002
	Roy's Largest Root	.364	11.287[b]	1.000	31.000	.002
peasscore * PGY	Pillai's Trace	.145	2.635[b]	2.000	31.000	.088
	Wilks' Lambda	.855	2.635[b]	2.000	31.000	.088
	Hotelling's Trace	.170	2.635[b]	2.000	31.000	.088
	Roy's Largest Root	.170	2.635[b]	2.000	31.000	.088
peasscore * study	Pillai's Trace	.098	3.354[b]	1.000	31.000	.077
	Wilks' Lambda	.902	3.354[b]	1.000	31.000	.077
	Hotelling's Trace	.108	3.354[b]	1.000	31.000	.077
	Roy's Largest Root	.108	3.354[b]	1.000	31.000	.077
peasscore * exposure * PGY	Pillai's Trace	.061	1.005[b]	2.000	31.000	.378
	Wilks' Lambda	.939	1.005[b]	2.000	31.000	.378
	Hotelling's Trace	.065	1.005[b]	2.000	31.000	.378
	Roy's Largest Root	.065	1.005[b]	2.000	31.000	.378
peasscore * exposure * study	Pillai's Trace	.170	6.345[b]	1.000	31.000	.017
	Wilks' Lambda	.830	6.345[b]	1.000	31.000	.017
	Hotelling's Trace	.205	6.345[b]	1.000	31.000	.017
	Roy's Largest Root	.205	6.345[b]	1.000	31.000	.017
peasscore * PGY * study	Pillai's Trace	.064	2.125[b]	1.000	31.000	.155
	Wilks' Lambda	.936	2.125[b]	1.000	31.000	.155
	Hotelling's Trace	.069	2.125[b]	1.000	31.000	.155
	Roy's Largest Root	.069	2.125[b]	1.000	31.000	.155
peasscore * exposure * PGY * study	Pillai's Trace	.000	.[b]	.000	.000	.
	Wilks' Lambda	1.000	.[b]	.000	31.000	.
	Hotelling's Trace	.000	.[b]	.000	2.000	.
	Roy's Largest Root	.000	.000[b]	1.000	30.000	1.000

a. Design: Intercept + exposure + PGY + study + exposure * PGY + exposure * study + PGY * study + exposure * PGY * study
Within Subjects Design: peasscore

b. Exact statistic

Mauchly's Test of Sphericity[a]

Measure: MEASURE_1

Within Subjects Effect	Mauchly's W	Approx. Chi-Square	df	Sig.	Epsilon[b]		
					Greenhouse-Geisser	Huynh-Feldt	Lower-bound
peasscore	1.000	.000	0	.	1.000	1.000	1.000

Tests the null hypothesis that the error covariance matrix of the orthonormalized transformed dependent variables is proportional to an identity matrix.

a. Design: Intercept + exposure + PGY + study + exposure * PGY + exposure * study + PGY * study + exposure * PGY * study
Within Subjects Design: peasscore

b. May be used to adjust the degrees of freedom for the averaged tests of significance. Corrected tests are displayed in the Tests of Within-Subjects Effects table.

Tests of Within-Subjects Effects

Measure: MEASURE_1

Source		Type III Sum of Squares	df	Mean Square	F	Sig.
peasscore	Sphericity Assumed	208.896	1	208.896	10.461	.003
	Greenhouse-Geisser	208.896	1.000	208.896	10.461	.003
	Huynh-Feldt	208.896	1.000	208.896	10.461	.003
	Lower-bound	208.896	1.000	208.896	10.461	.003
peasscore * exposure	Sphericity Assumed	225.388	1	225.388	11.287	.002
	Greenhouse-Geisser	225.388	1.000	225.388	11.287	.002
	Huynh-Feldt	225.388	1.000	225.388	11.287	.002
	Lower-bound	225.388	1.000	225.388	11.287	.002
peasscore * PGY	Sphericity Assumed	105.250	2	52.625	2.635	.088
	Greenhouse-Geisser	105.250	2.000	52.625	2.635	.088
	Huynh-Feldt	105.250	2.000	52.625	2.635	.088
	Lower-bound	105.250	2.000	52.625	2.635	.088

Tests of Within-Subjects Effects

Measure: MEASURE_1

Source		Type III Sum of Squares	df	Mean Square	F	Sig.
peasscore * study	Sphericity Assumed	66.965	1	66.965	3.354	.077
	Greenhouse-Geisser	66.965	1.000	66.965	3.354	.077
	Huynh-Feldt	66.965	1.000	66.965	3.354	.077
	Lower-bound	66.965	1.000	66.965	3.354	.077
peasscore * exposure * PGY	Sphericity Assumed	40.137	2	20.069	1.005	.378
	Greenhouse-Geisser	40.137	2.000	20.069	1.005	.378
	Huynh-Feldt	40.137	2.000	20.069	1.005	.378
	Lower-bound	40.137	2.000	20.069	1.005	.378
peasscore * exposure * study	Sphericity Assumed	126.707	1	126.707	6.345	.017
	Greenhouse-Geisser	126.707	1.000	126.707	6.345	.017
	Huynh-Feldt	126.707	1.000	126.707	6.345	.017
	Lower-bound	126.707	1.000	126.707	6.345	.017
peasscore * PGY * study	Sphericity Assumed	42.438	1	42.438	2.125	.155
	Greenhouse-Geisser	42.438	1.000	42.438	2.125	.155
	Huynh-Feldt	42.438	1.000	42.438	2.125	.155
	Lower-bound	42.438	1.000	42.438	2.125	.155
peasscore * exposure * PGY * study	Sphericity Assumed	.000	0	.	.	.
	Greenhouse-Geisser	.000	.000	.	.	.
	Huynh-Feldt	.000	.000	.	.	.
	Lower-bound	.000	.000	.	.	.
Error(peasscore)	Sphericity Assumed	619.019	31	19.968		
	Greenhouse-Geisser	619.019	31.000	19.968		
	Huynh-Feldt	619.019	31.000	19.968		
	Lower-bound	619.019	31.000	19.968		

Tests of Within-Subjects Contrasts

Measure: MEASURE_1

Source	peasscore	Type III Sum of Squares	df	Mean Square	F	Sig.
peasscore	Linear	208.896	1	208.896	10.461	.003
peasscore * exposure	Linear	225.388	1	225.388	11.287	.002
peasscore * PGY	Linear	105.250	2	52.625	2.635	.088
peasscore * study	Linear	66.965	1	66.965	3.354	.077
peasscore * exposure * PGY	Linear	40.137	2	20.069	1.005	.378
peasscore * exposure * study	Linear	126.707	1	126.707	6.345	.017
peasscore * PGY * study	Linear	42.438	1	42.438	2.125	.155
peasscore * exposure * PGY * study	Linear	.000	0	.	.	.
Error(peasscore)	Linear	619.019	31	19.968		

Tests of Between-Subjects Effects

Measure: MEASURE_1

Transformed Variable: Average

Source	Type III Sum of Squares	df	Mean Square	F	Sig.
Intercept	210391.9	1	210391.9	3256.837	.000
exposure	10.994	1	10.994	.170	.683
PGY	558.686	2	279.343	4.324	.022
study	293.378	1	293.378	4.541	.041
exposure * PGY	945.499	2	472.750	7.318	.002
exposure * study	42.677	1	42.677	.661	.423
PGY * study	31.084	1	31.084	.481	.493
exposure * PGY * study	.000	0	.	.	.
Error	2002.603	31	64.600		

Profile Plots

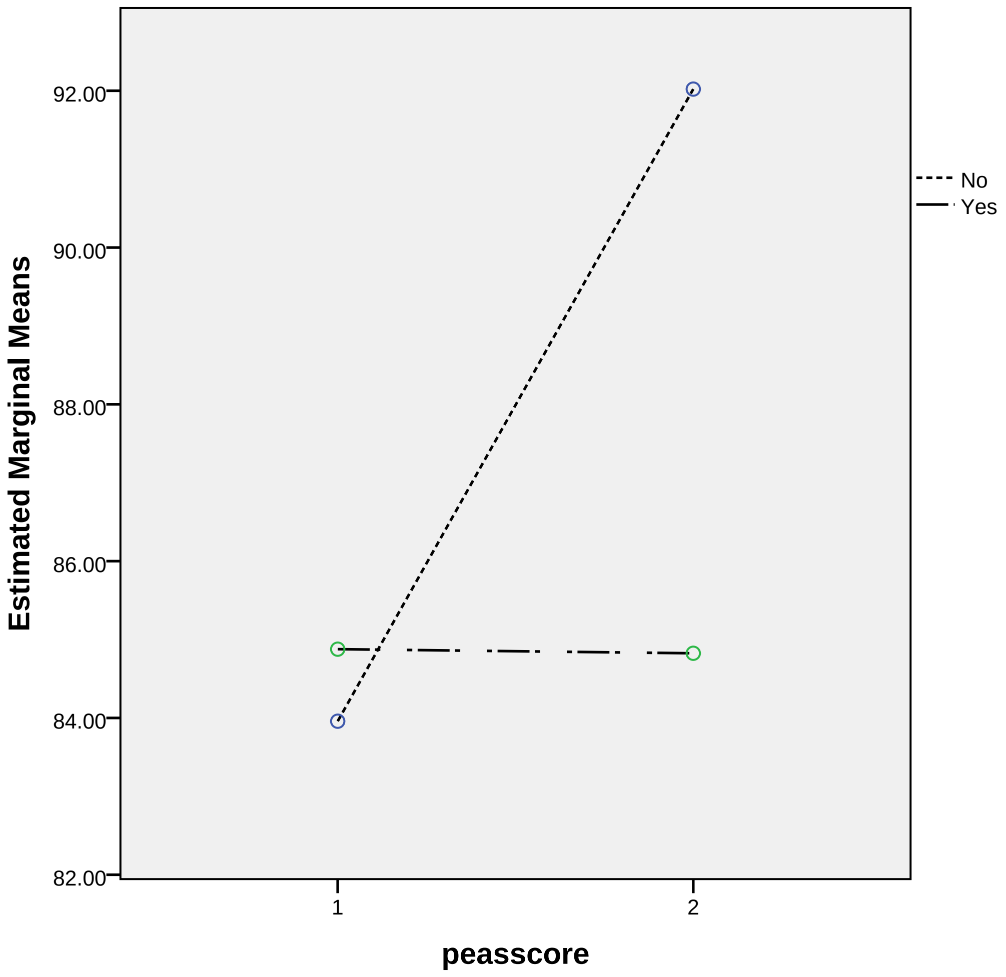
92.00
90.00
88.00
86.00
84.00
82.00
Estimated Marginal Means
1
2
peasscore
No
Yes

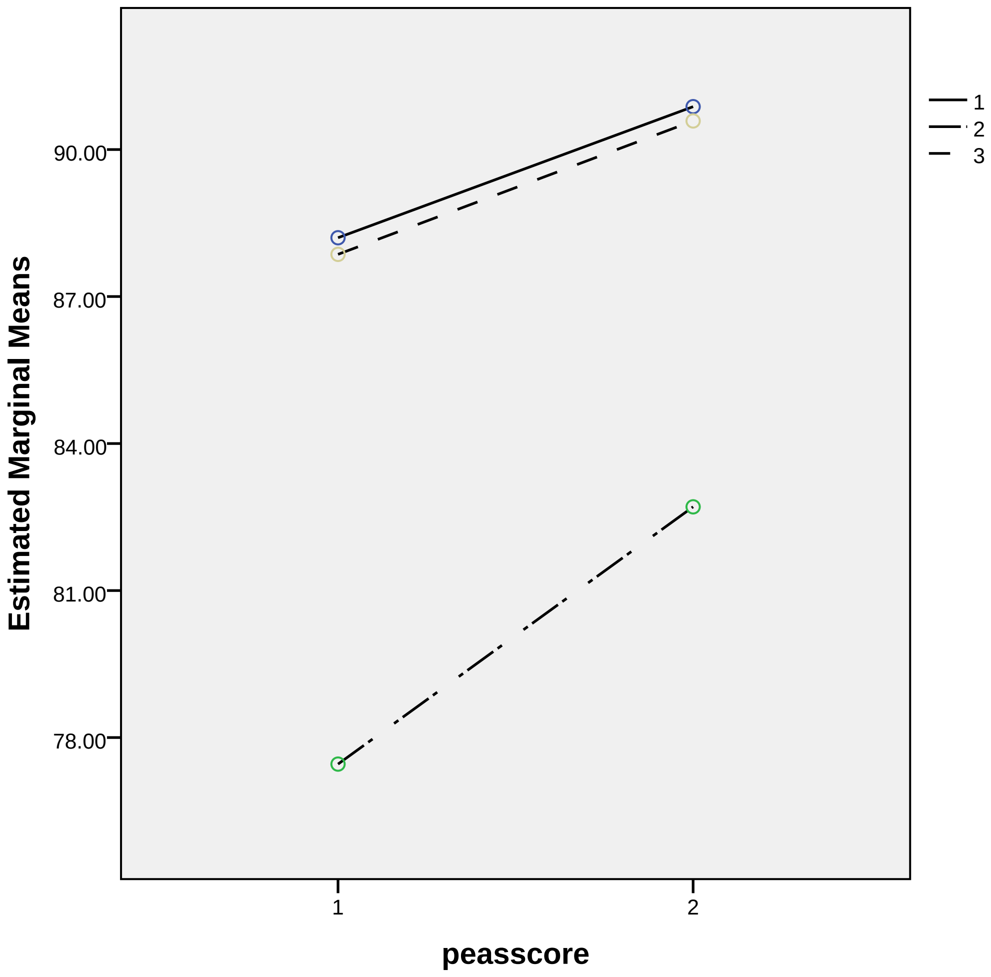

Estimated Marginal Means
90.00
87.00
84.00
81.00
78.00
1
2
peasscore
1
2
3

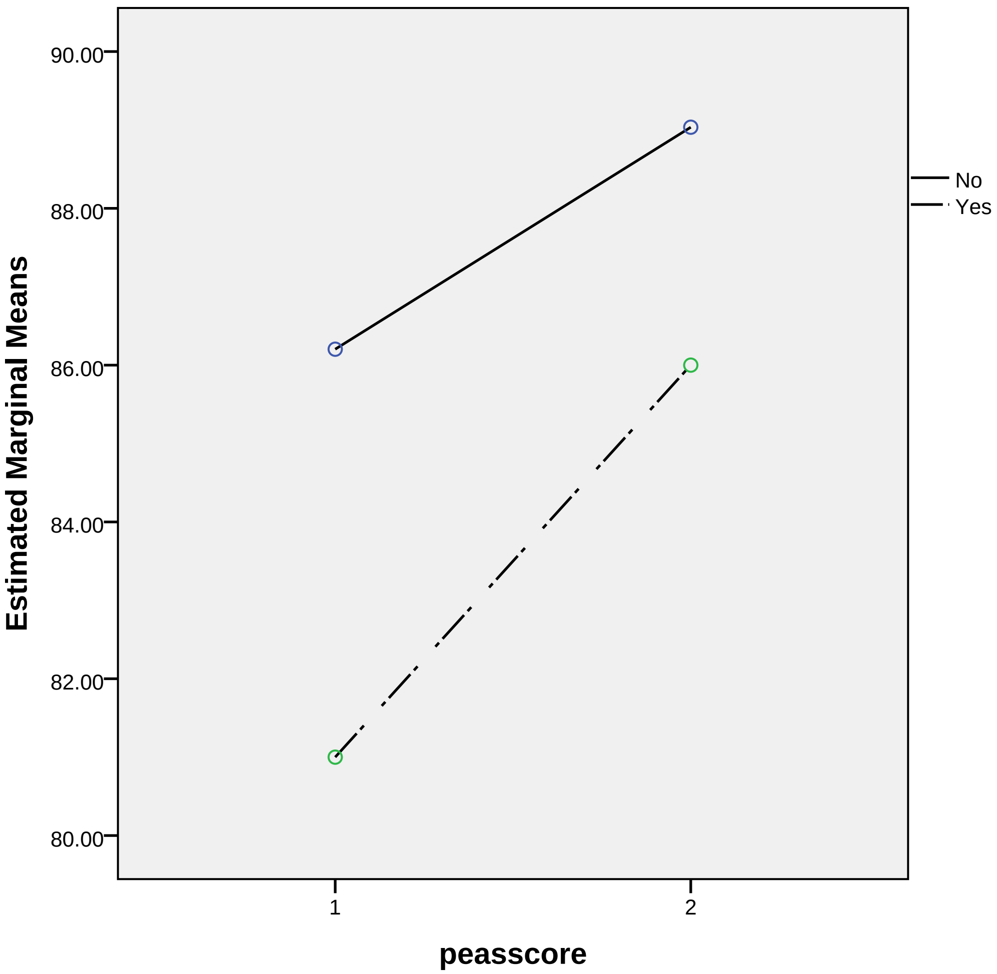


```
T-TEST GROUPS=age_g(1 2)
  /MISSING=ANALYSIS
  /VARIABLES=PEAS_diff
  /CRITERIA=CI(.95).
```

T-Test

```
[DataSet1] C:\Users\user\Desktop\Analysis Payanname\pre-post analysis.sav
```

Group Statistics

		N	Mean	Std. Deviation	Std. Error Mean
PEAS_diff	=<35	21	2.3810	6.88096	1.50155
	>35	19	1.6842	6.87227	1.57661

Independent Samples Test

		Levene's Test for Equality of Variances		t-test for Equality of Means			
		F	Sig.	t	df	Sig. (2-tailed)	Mean Difference
PEAS_diff	Equal variances assumed	.105	.747	.320	38	.751	.69674
	Equal variances not assumed			.320	37.612	.751	.69674

Independent Samples Test

		t-test for Equality of Means		
		Std. Error Difference	95% Confidence Interval of the Difference	
			Lower	Upper
PEAS_diff	Equal variances assumed	2.17737	-3.71112	5.10460
	Equal variances not assumed	2.17723	-3.71232	5.10581

```
T-TEST GROUPS=Clin_exp_grp(1 2)
 /MISSING=ANALYSIS
 /VARIABLES=PEAS_diff
 /CRITERIA=CI(.95).
```

T-Test

Group Statistics

		N	Mean	Std. Deviation	Std. Error Mean
PEAS_diff	=<4	20	3.3000	7.65781	1.71234
	>4	18	.8889	6.05746	1.42776

Independent Samples Test

		Levene's Test for Equality of Variances		t-test for Equality of Means			
		F	Sig.	t	df	Sig. (2-tailed)	Mean Difference
PEAS_diff	Equal variances assumed	.542	.466	1.068	36	.293	2.41111
	Equal variances not assumed			1.081	35.451	.287	2.41111

Independent Samples Test

		t-test for Equality of Means		
		Std. Error Difference	95% Confidence Interval of the Difference	
			Lower	Upper
PEAS_diff	Equal variances assumed	2.25742	-2.16714	6.98936
	Equal variances not assumed	2.22948	-2.11292	6.93514

```
T-TEST GROUPS=Gender(1 2)
 /MISSING=ANALYSIS
 /VARIABLES=PEAS_diff
 /CRITERIA=CI(.95).
```

T-Test

Group Statistics

	Gender	N	Mean	Std. Deviation	Std. Error Mean
PEAS_diff	Female	27	.9630	6.59534	1.26927
	Male	13	4.3077	6.90875	1.91614

Independent Samples Test

		Levene's Test for Equality of Variances		t-test for Equality of Means			
		F	Sig.	t	df	Sig. (2-tailed)	Mean Difference
PEAS_diff	Equal variances assumed	.005	.947	-1.480	38	.147	-3.34473
	Equal variances not assumed			-1.455	22.814	.159	-3.34473

Independent Samples Test

		t-test for Equality of Means		
		Std. Error Difference	95% Confidence Interval of the Difference	
			Lower	Upper
PEAS_diff	Equal variances assumed	2.26040	-7.92067	1.23121
	Equal variances not assumed	2.29840	-8.10148	1.41202

```
T-TEST GROUPS=study(1 2)
 /MISSING=ANALYSIS
 /VARIABLES=PEAS_diff
 /CRITERIA=CI(.95).
```

T-Test

Group Statistics

		N	Mean	Std. Deviation	Std. Error Mean
PEAS_diff	No	36	1.7222	6.38127	1.06355
	Yes	4	5.0000	10.61446	5.30723

Independent Samples Test

		Levene's Test for Equality of Variances		t-test for Equality of Means			
		F	Sig.	t	df	Sig. (2-tailed)	Mean Difference
PEAS_diff	Equal variances assumed	.398	.532	-.913	38	.367	-3.27778
	Equal variances not assumed			-.606	3.245	.585	-3.27778

Independent Samples Test

		t-test for Equality of Means		
		Std. Error Difference	95% Confidence Interval of the Difference	
			Lower	Upper
PEAS_diff	Equal variances assumed	3.59013	-10.5456	3.99007
	Equal variances not assumed	5.41274	-19.7898	13.23422

```
T-TEST GROUPS=exposure(1 2)
 /MISSING=ANALYSIS
 /VARIABLES=PEAS_diff
 /CRITERIA=CI(.95).
```

T-Test

Group Statistics

		N	Mean	Std. Deviation	Std. Error Mean
PEAS_diff	No	10	5.4000	7.73448	2.44586
	Yes	30	.9333	6.19752	1.13151

Independent Samples Test

		Levene's Test for Equality of Variances		t-test for Equality of Means			
		F	Sig.	t	df	Sig. (2-tailed)	Mean Difference
PEAS_diff	Equal variances assumed	.733	.397	1.855	38	.071	4.46667
	Equal variances not assumed			1.657	13.079	.121	4.46667

Independent Samples Test

		t-test for Equality of Means		
		Std. Error Difference	95% Confidence Interval of the Difference	
			Lower	Upper
PEAS_diff	Equal variances assumed	2.40778	-.40764	9.34097
	Equal variances not assumed	2.69491	-1.35177	10.28510

```
ONEWAY PEAS_diff BY PGY
 /MISSING ANALYSIS.
```

```
ONEWAY PEAS_diff BY PGY
 /MISSING ANALYSIS
 /POSTHOC=BTUKEY ALPHA(0.05).
```

Oneway

ANOVA

PEAS_diff

	Sum of Squares	df	Mean Square	F	Sig.
Between Groups	32.757	2	16.379	.343	.712
Within Groups	1769.143	37	47.815		
Total	1801.900	39			

Post Hoc Tests

Homogeneous Subsets

PEAS_diff

Tukey B[a,b]

	N	Subset for alpha = 0.05
		1
3	14	.8571
1	14	2.4286
2	12	3.0000

Means for groups in homogeneous subsets are displayed.

a. Uses Harmonic Mean Sample Size = 13.263.

b. The group sizes are unequal. The harmonic mean of the group sizes is used. Type I error levels are not guaranteed.

```
T-TEST GROUPS=study(1 2)
 /MISSING=ANALYSIS
 /VARIABLES=PEAS_pre
 /CRITERIA=CI(.95).
```

Base line **

Group Statistics

		N	Mean	Std. Deviation	Std. Error Mean
PEAS score baseline	No	36	87.8333	8.38877	1.39813
	Yes	4	79.2500	6.07591	3.03795

Independent Samples Test

		Levene's Test for Equality of Variances		t-test for Equality of Means		
		F	Sig.	t	df	Sig. (2-tailed)
PEAS score baseline	Equal variances assumed	.259	.614	1.979	38	.055
	Equal variances not assumed			2.567	4.389	.057

Independent Samples Test

		t-test for Equality of Means			
		Mean Difference	Std. Error Difference	95% Confidence Interval of the Difference	
				Lower	Upper
PEAS score baseline	Equal variances assumed	8.58333	4.33751	-.19749	17.36416
	Equal variances not assumed	8.58333	3.34424	-.38638	17.55305

```
T-TEST GROUPS=Gender(1 2)
 /MISSING=ANALYSIS
 /VARIABLES=PEAS_pre
 /CRITERIA=CI(.95).
```

T-Test

Group Statistics

	Gender	N	Mean	Std. Deviation	Std. Error Mean
PEAS score baseline	Female	27	88.1111	9.22093	1.77457
	Male	13	84.6154	6.58962	1.82763

Independent Samples Test

		Levene's Test for Equality of Variances		t-test for Equality of Means		
		F	Sig.	t	df	Sig. (2-tailed)
PEAS score baseline	Equal variances assumed	1.780	.190	1.221	38	.229
	Equal variances not assumed			1.372	32.117	.179

Independent Samples Test

		t-test for Equality of Means			
		Mean Difference	Std. Error Difference	95% Confidence Interval of the Difference	
				Lower	Upper
PEAS score baseline	Equal variances assumed	3.49573	2.86223	-2.29856	9.29001
	Equal variances not assumed	3.49573	2.54742	-1.69245	8.68390

```
T-TEST GROUPS=Clin_exp_grp(1 2)
 /MISSING=ANALYSIS
 /VARIABLES=PEAS_pre
 /CRITERIA=CI(.95).
```

T-Test

Group Statistics

		N	Mean	Std. Deviation	Std. Error Mean
PEAS score baseline	=<4	20	85.6000	8.37540	1.87280
	>4	18	88.7222	9.09302	2.14325

Independent Samples Test

		Levene's Test for Equality of Variances		t-test for Equality of Means		
		F	Sig.	t	df	Sig. (2-tailed)
PEAS score baseline	Equal variances assumed	.572	.455	-1.102	36	.278
	Equal variances not assumed			-1.097	34.747	.280

Independent Samples Test

		t-test for Equality of Means			
		Mean Difference	Std. Error Difference	95% Confidence Interval of the Difference	
				Lower	Upper
PEAS score baseline	Equal variances assumed	-3.12222	2.83360	-8.86903	2.62459
	Equal variances not assumed	-3.12222	2.84620	-8.90183	2.65738

```
T-TEST GROUPS=age_g(1 2)
 /MISSING=ANALYSIS
 /VARIABLES=PEAS_pre
 /CRITERIA=CI(.95).
```

T-Test

Group Statistics

		N	Mean	Std. Deviation	Std. Error Mean
PEAS score baseline	=<35	21	85.8571	9.29670	2.02871
	>35	19	88.2105	7.65636	1.75649

Independent Samples Test

		Levene's Test for Equality of Variances		t-test for Equality of Means		
		F	Sig.	t	df	Sig. (2-tailed)
PEAS score baseline	Equal variances assumed	.373	.545	-.868	38	.391
	Equal variances not assumed			-.877	37.691	.386

Independent Samples Test

		t-test for Equality of Means			
		Mean Difference	Std. Error Difference	95% Confidence Interval of the Difference	
				Lower	Upper
PEAS score baseline	Equal variances assumed	-2.35338	2.70997	-7.83944	3.13267
	Equal variances not assumed	-2.35338	2.68345	-7.78721	3.08044

```
T-TEST GROUPS=exposure(1 2)
 /MISSING=ANALYSIS
 /VARIABLES=PEAS_pre
 /CRITERIA=CI(.95).
```

T-Test

Group Statistics

		N	Mean	Std. Deviation	Std. Error Mean
PEAS score baseline	No	10	86.0000	10.77033	3.40588
	Yes	30	87.3000	7.83955	1.43130

Independent Samples Test

		Levene's Test for Equality of Variances		t-test for Equality of Means		
		F	Sig.	t	df	Sig. (2-tailed)
PEAS score baseline	Equal variances assumed	.872	.356	-.413	38	.682
	Equal variances not assumed			-.352	12.340	.731

Independent Samples Test

		t-test for Equality of Means			
		Mean Difference	Std. Error Difference	95% Confidence Interval of the Difference	
				Lower	Upper
PEAS score baseline	Equal variances assumed	-1.30000	3.14910	-7.67502	5.07502
	Equal variances not assumed	-1.30000	3.69440	-9.32487	6.72487

```
T-TEST GROUPS=study(1 2)
 /MISSING=ANALYSIS
 /VARIABLES=PEAS_pre
 /CRITERIA=CI(.95).
```

T-Test

Group Statistics

		N	Mean	Std. Deviation	Std. Error Mean
PEAS score baseline	No	36	87.8333	8.38877	1.39813
	Yes	4	79.2500	6.07591	3.03795

Independent Samples Test

		Levene's Test for Equality of Variances		t-test for Equality of Means		
		F	Sig.	t	df	Sig. (2-tailed)
PEAS score baseline	Equal variances assumed	.259	.614	1.979	38	.055
	Equal variances not assumed			2.567	4.389	.057

Independent Samples Test

		t-test for Equality of Means			
		Mean Difference	Std. Error Difference	95% Confidence Interval of the Difference	
				Lower	Upper
PEAS score baseline	Equal variances assumed	8.58333	4.33751	-.19749	17.36416
	Equal variances not assumed	8.58333	3.34424	-.38638	17.55305

Oneway

Descriptives

PEAS score baseline

	N	Mean	Std. Deviation	Std. Error	95% Confidence Interval for Mean		Minimum	Maximum
					Lower Bound	Upper Bound		
1	14	87.6429	8.68781	2.32191	82.6267	92.6590	73.00	101.00
2	12	83.4167	9.38527	2.70929	77.4535	89.3798	69.00	94.00
3	14	89.3571	7.07767	1.89159	85.2706	93.4437	79.00	100.00
Total	40	86.9750	8.53195	1.34902	84.2463	89.7037	69.00	101.00

ANOVA

PEAS score baseline

	Sum of Squares	df	Mean Square	F	Sig.
Between Groups	237.630	2	118.815	1.690	.198
Within Groups	2601.345	37	70.307		
Total	2838.975	39			

```
ONEWAY PEAS_diff BY PGY
  /STATISTICS DESCRIPTIVES
  /MISSING ANALYSIS.
```

Oneway

Descriptives

PEAS_diff

	N	Mean	Std. Deviation	Std. Error	95% Confidence Interval for Mean Lower Bound	95% Confidence Interval for Mean Upper Bound	Minimum	Maximum
1	14	2.4286	6.42967	1.71840	-1.2838	6.1410	-6.00	13.00
2	12	3.0000	4.02266	1.16124	.4441	5.5559	-1.00	10.00
3	14	.8571	9.00305	2.40617	-4.3411	6.0554	-12.00	18.00
Total	40	2.0500	6.79725	1.07474	-.1239	4.2239	-12.00	18.00

ANOVA

PEAS_diff

	Sum of Squares	df	Mean Square	F	Sig.
Between Groups	32.757	2	16.379	.343	.712
Within Groups	1769.143	37	47.815		
Total	1801.900	39			

```
SET SUMMARY=None TOLERANCE=1 CELLSBREAK=10000 TABLERENDER=light ROWSBREAK=100
TLook=None DIGITGROUPING=No Small=0.0001 TFit=Both.
```

```
COMPUTE pre_Sub_1=SUM (q1,q2,q3,q4,q5,q6,q7,q8,q9,q10,q11,q12,q13).
EXECUTE.
COMPUTE pre_Sub_2=SUM(q14,q15,q16,q17,q18,q19,q20,q21,q22,q23,q24,q25,q26,q27,q28,q29,q30,q31).
EXECUTE.
COMPUTE post_Sub_1=SUM(q1p,q2p,q3p,q4p,q5p,q6p,q7p,q8p,q9p,q10p,q11p,q12p,q13p).
EXECUTE.
COMPUTE post_Sub_2=SUM(q14p,q15p,q16p,q17p,q18p,q19p,q20p,q21p,q22p,q23p,q24p,q25p,q26p,q27p,q28p,
    q29p,q30p,q31p).
EXECUTE.
```

Descriptive Statistics

	N	Mean	Std. Deviation
pre_Sub_1	40	36.7000	5.60769
post_Sub_1	40	37.8500	5.06648
pre_Sub_2	40	50.2750	4.17555
post_Sub_2	40	51.1750	3.50741
Valid N (listwise)	40		

Correlations

		post_Sub_1	post_Sub_2
pre_Sub_1		.654	.385
		.000	.014
		40	40
pre_Sub_2		.143	.560
		.380	.000
		40	40

```
COMPUTE Adj_pre_sub_1=pre_Sub_1+ (1-.654)*(3
```

```
6.7-pre_Sub_1).
EXECUTE.
```

```
COMPUTE Adj_pre_sub_2=pre_Sub_2+ (1-.560)*(5
0.27-pre_Sub_2).
EXECUTE.
```

Descriptives

Descriptive Statistics

	N	Minimum	Maximum	Mean	Std. Deviation
pre_Sub_1	40	24.00	44.00	36.7000	5.60769
Adj_pre_sub_1	40	28.39	41.47	36.7000	3.66743
pre_Sub_2	40	41.00	57.00	50.2750	4.17555
Adj_pre_sub_2	40	45.08	54.04	50.2728	2.33831
Valid N (listwise)	40				

```
 COMPUTE Adj_diff_sub_1=Adj_pre_sub_1-post_Sub_1.
EXECUTE.
COMPUTE Adj_diff_sub_2=Adj_pre_sub_2-post_Sub_2.
EXECUTE.
DESCRIPTIVES VARIABLES=Adj_diff_sub_1 Adj_diff_sub_2
  /STATISTICS=MEAN STDDEV MIN MAX.
```

Descriptives

Descriptive Statistics

	N	Minimum	Maximum	Mean	Std. Deviation
Adj_diff_sub_1	40	-12.41	3.55	-1.1500	3.85106
Adj_diff_sub_2	40	-7.12	8.24	-.9022	2.92877
Valid N (listwise)	40				

```
T-TEST PAIRS=Adj_pre_sub_1 Adj_pre_sub_2 WITH post_Sub_1 post_Sub_2 (PAIRED)
 /CRITERIA=CI(.9500)
 /MISSING=ANALYSIS.
```

T-Test

Paired Samples Statistics

		Mean	N	Std. Deviation	Std. Error Mean
Pair 1	Adj_pre_sub_1	36.7000	40	3.66743	.57987
	post_Sub_1	37.8500	40	5.06648	.80108
Pair 2	Adj_pre_sub_2	50.2728	40	2.33831	.36972
	post_Sub_2	51.1750	40	3.50741	.55457

Paired Samples Correlations

		N	Correlation	Sig.
Pair 1	Adj_pre_sub_1 & post_Sub_1	40	.654	.000
Pair 2	Adj_pre_sub_2 & post_Sub_2	40	.560	.000

Paired Samples Test

		Paired Differences			
					95% Confidence ...
		Mean	Std. Deviation	Std. Error Mean	Lower
Pair 1	Adj_pre_sub_1 - post_Sub_1	-1.15000	3.85106	.60891	-2.38163
Pair 2	Adj_pre_sub_2 - post_Sub_2	-.90220	2.92877	.46308	-1.83887

Paired Samples Test

		Paired ...			
		95% Confidence Interval of the ...			
		Upper	t	df	Sig. (2-tailed)
Pair 1	Adj_pre_sub_1 - post_Sub_1	.08163	-1.889	39	.066
Pair 2	Adj_pre_sub_2 - post_Sub_2	.03447	-1.948	39	.059

```
NPAR TESTS
 /K-S(NORMAL)=Adj_pre_sub_1 Adj_pre_sub_2 post_Sub_1 post_Sub_2
 /STATISTICS DESCRIPTIVES
 /MISSING ANALYSIS.
```

NPar Tests

Descriptive Statistics

	N	Mean	Std. Deviation	Minimum	Maximum
Adj_pre_sub_1	40	36.7000	3.66743	28.39	41.47
Adj_pre_sub_2	40	50.2728	2.33831	45.08	54.04
post_Sub_1	40	37.8500	5.06648	30.00	48.00
post_Sub_2	40	51.1750	3.50741	43.00	58.00

One-Sample Kolmogorov-Smirnov Test

		Adj_pre_sub_1	Adj_pre_sub_2	post_Sub_1	post_Sub_2
N		40	40	40	40
Normal Parameters[a,b]	Mean	36.7000	50.2728	37.8500	51.1750
	Std. Deviation	3.66743	2.33831	5.06648	3.50741
Most Extreme Differences	Absolute	.167	.110	.126	.095
	Positive	.096	.109	.126	.095
	Negative	-.167	-.110	-.096	-.087
Test Statistic		.167	.110	.126	.095
Asymp. Sig. (2-tailed)		.007[c]	.200[c,d]	.108[c]	.200[c,d]

a. Test distribution is Normal.

b. Calculated from data.

c. Lilliefors Significance Correction.

d. This is a lower bound of the true significance.

```
NPAR TESTS
 /WILCOXON=Adj_pre_sub_1 Adj_pre_sub_2 WITH post_Sub_1 post_Sub_2 (PAIRED)
 /MISSING ANALYSIS.
```

NPar Tests

Wilcoxon Signed Ranks Test

Ranks

		N	Mean Rank	Sum of Ranks
post_Sub_1 - Adj_pre_sub_1	Negative Ranks	21[a]	15.19	319.00
	Positive Ranks	19[b]	26.37	501.00
	Ties	0[c]		
	Total	40		
post_Sub_2 - Adj_pre_sub_2	Negative Ranks	13[d]	19.15	249.00
	Positive Ranks	27[e]	21.15	571.00
	Ties	0[f]		
	Total	40		

a. post_Sub_1 < Adj_pre_sub_1

b. post_Sub_1 > Adj_pre_sub_1

c. post_Sub_1 = Adj_pre_sub_1

d. post_Sub_2 < Adj_pre_sub_2

e. post_Sub_2 > Adj_pre_sub_2

f. post_Sub_2 = Adj_pre_sub_2

Test Statistics[a]

	post_Sub_1 - Adj_pre_sub_1	post_Sub_2 - Adj_pre_sub_2
Z	-1.223[b]	-2.164[b]
Asymp. Sig. (2-tailed)	**.221**	.030

a. Wilcoxon Signed Ranks Test

b. Based on negative ranks.

```
CROSSTABS
 /TABLES=exposure BY study
 /FORMAT=AVALUE TABLES
 /STATISTICS=CHISQ
 /CELLS=COUNT ROW COLUMN
 /COUNT ROUND CELL.
```

Crosstabs

Case Processing Summary

	Cases					
	Valid		Missing		Total	
	N	Percent	N	Percent	N	Percent
*	40	100.0%	0	0.0%	40	100.0%

*** Crosstabulation**

			No	Yes	Total
	No	Count	9	1	10
		% within	90.0%	10.0%	100.0%
		% within	25.0%	25.0%	25.0%
	Yes	Count	27	3	30
		% within	90.0%	10.0%	100.0%
		% within	75.0%	75.0%	75.0%
Total		Count	36	4	40
		% within	90.0%	10.0%	100.0%
		% within	100.0%	100.0%	100.0%

Chi-Square Tests

	Value	df	Asymptotic Significance (2-sided)	Exact Sig. (2-sided)	Exact Sig. (1-sided)
Pearson Chi-Square	.000[a]	1	1.000		
Continuity Correction[b]	.000	1	1.000		
Likelihood Ratio	.000	1	1.000		
Fisher's Exact Test				1.000	.700
Linear-by-Linear Association	.000	1	1.000		
N of Valid Cases	40				

a. 2 cells (50.0%) have expected count less than 5. The minimum expected count is 1.00.

b. Computed only for a 2x2 table

```
CROSSTABS
  /TABLES=age_g Clin_exp_grp BY study
  /FORMAT=AVALUE TABLES
  /STATISTICS=CHISQ
  /CELLS=COUNT ROW COLUMN
  /COUNT ROUND CELL.
```

Crosstabs

گروه سنی * سابقه مطالعه در مورد بیماران انتهایی

Crosstab

		No	Yes	Total
=<35	Count	21	0	21
	% within	100.0%	0.0%	100.0%
	% within	58.3%	0.0%	52.5%
>35	Count	15	4	19
	% within	78.9%	21.1%	100.0%
	% within	41.7%	100.0%	47.5%
Total	Count	36	4	40
	% within	90.0%	10.0%	100.0%
	% within	100.0%	100.0%	100.0%

Chi-Square Tests

	Value	df	Asymptotic Significance (2-sided)	Exact Sig. (2-sided)	Exact Sig. (1-sided)
Pearson Chi-Square	4.912[a]	1	.027		
Continuity Correction[b]	2.852	1	.091		
Likelihood Ratio	6.450	1	.011		
Fisher's Exact Test				.042	.042
Linear-by-Linear Association	4.789	1	.029		
N of Valid Cases	40				

a. 2 cells (50.0%) have expected count less than 5. The minimum expected count is 1.90.

b. Computed only for a 2x2 table

گروه سابقه کار بالینی * سابقه مطالعه در مورد بیماران انتهایی

Crosstab

			No	Yes	Total
	=<4	Count	19	1	20
		% within	95.0%	5.0%	100.0%
		% within	55.9%	25.0%	52.6%
	>4	Count	15	3	18
		% within	83.3%	16.7%	100.0%
		% within	44.1%	75.0%	47.4%
Total		Count	34	4	38
		% within	89.5%	10.5%	100.0%
		% within	100.0%	100.0%	100.0%

Chi-Square Tests

	Value	df	Asymptotic Significance (2-sided)	Exact Sig. (2-sided)	Exact Sig. (1-sided)
Pearson Chi-Square	1.369[a]	1	.242		
Continuity Correction[b]	.411	1	.522		
Likelihood Ratio	1.413	1	.235		
Fisher's Exact Test				.328	.263
Linear-by-Linear Association	1.333	1	.248		
N of Valid Cases	38				

a. 2 cells (50.0%) have expected count less than 5. The minimum expected count is 1.89.

b. Computed only for a 2x2 table

```
T-TEST PAIRS=Adj_pre_sub_1 pre_Sub_2 WITH post_Sub_1 post_Sub_2 (PAIRED)
 /CRITERIA=CI(.9500)
 /MISSING=ANALYSIS.
```

T-Test

Paired Samples Statistics

		Mean	N	Std. Deviation	Std. Error Mean
Pair 1	Adj_pre_sub_1	36.7000	40	3.66743	.57987
	post_Sub_1	37.8500	40	5.06648	.80108
Pair 2	pre_Sub_2	50.2750	40	4.17555	.66021
	post_Sub_2	51.1750	40	3.50741	.55457

Paired Samples Correlations

		N	Correlation	Sig.
Pair 1	Adj_pre_sub_1 & post_Sub_1	40	.654	.000
Pair 2	pre_Sub_2 & post_Sub_2	40	.560	.000

Paired Samples Test

		Paired Differences			
					95% Confidence ...
		Mean	Std. Deviation	Std. Error Mean	Lower
Pair 1	Adj_pre_sub_1 - post_Sub_1	-1.15000	3.85106	.60891	-2.38163
Pair 2	pre_Sub_2 - post_Sub_2	-.90000	3.65008	.57713	-2.06735

Paired Samples Test

		Paired ...			
		95% Confidence Interval of the ...			
		Upper	t	df	Sig. (2-tailed)
Pair 1	Adj_pre_sub_1 - post_Sub_1	.08163	-1.889	39	.066
Pair 2	pre_Sub_2 - post_Sub_2	.26735	-1.559	39	.127
