# Supplementary material for: Attitude toward end-of-life care in emergency medicine residents- can a short workshop make a difference?
Source: PLoS One. 2023 Jan 11;18(1):e0280229. doi: 10.1371/journal.pone.0280229 (PMC9833511; doi:10.1371/journal.pone.0280229)
Supplement: S1 Questionaire — (PDF) [file pone.0280229.s002.pdf]

پرسشنامه نگرش حرفه ای به پایان زندگی در بیماران مبتلا به شرایط نهایتا کشنده (بیماران محتضر)

Professional End-of-Life Attitude Scale Test Questions

- نام و نام خانوادگی (یا نام مستعار) : ____________________
- جنسیت : ۱- خانم ۲- آقا
- سن : __________
- سال رزیدنتی: ___________
- سابقه کار بالینی پس از فارغ التحصیلی: __________ سال و ___________ ماه
- سابقه برخورد با بیمار مبتلا به بیماری انتهایی در نزدیکان: ۱- نداشته ام ۲- داشته ام
- سابقه مطالعه در مورد مراقبتهای بیماران End-stage در اورژانس:

۱- نداشته ام ۲- داشته ام، منابع استفاده شده: ______________________________________

	دور مناسبترین گزینه خط بکشید						
۱	من ترجیح میدهم مراقبت از بیمارانی را بعهده بگیرم که بتوانم برای آنها کاری انجام دهم تا بیمارانی که بعلت بیماری غیر قابل درمان بزودی خواهند مرد.	کاملا موافق ۱	موافق ۲	نه موافق نه مخالف ۳	مخالف ۴	بشدت مخالف ۵	اصلا نظری ندارم ۰
۲	وقتی بیماری فوت می کند در صورت اندوهگین بودن یکی از همکاران، آرام کردن (تسلی دادن) او اقدامی مناسب است.	کاملا موافق ۱	موافق ۲	نه موافق نه مخالف ۳	مخالف ۴	بشدت مخالف ۵	اصلا نظری ندارم ۰
۳	زمانیکه یکی از بیماران من فوت میکند احساس خشم میکنم.	کاملا موافق ۱	موافق ۲	نه موافق نه مخالف ۳	مخالف ۴	بشدت مخالف ۵	اصلا نظری ندارم ۰
۴	زمانیکه یکی از بیماران من فوت میکند احساس گناه میکنم.	کاملا موافق ۱	موافق ۲	نه موافق نه مخالف ۳	مخالف ۴	بشدت مخالف ۵	اصلا نظری ندارم ۰
۵	برقراری ارتباط با بیماری که بعلت بیماری غیر قابل درمان بزودی فوت خواهد کرد برای من مشکل است.	کاملا موافق ۱	موافق ۲	نه موافق نه مخالف ۳	مخالف ۴	بشدت مخالف ۵	اصلا نظری ندارم ۰
۶	ای کاش می شد که زمان بیشتری به بیمارانم که در حال مرگ هستند اختصاص بدهم.	کاملا موافق ۱	موافق ۲	نه موافق نه مخالف ۳	مخالف ۴	بشدت مخالف ۵	اصلا نظری ندارم ۰
۷	صحبت کردن در مورد اخذ رضایت برای عدم انجام اقدامات طبی در مراحل انتهایی بیماری، مانند دستور عدم احیا (DNR) یا دیالیز یا اتصال به ونتیلاتور وقتی که وضعیت بیمار پایدار است برای من مشکل می باشد.	کاملا موافق ۱	موافق ۲	نه موافق نه مخالف ۳	مخالف ۴	بشدت مخالف ۵	اصلا نظری ندارم ۰

۸	صحبت کردن در مورد اخذ رضایت برای عدم انجام اقدامات طبی در مراحل انتهایی بیماری، مانند دستور عدم احیا (DNR) یا دیالیز یا اتصال به ونتیلاتور وقتی که وضعیت بیمار پایدار است نامناسب است.	کاملا<br>موافق<br>۱	موافق<br>۲	نه موافق<br>نه مخالف<br>۳	مخالف<br>۴	بشدت<br>مخالف<br>۵	اصلا<br>نظری ندارم<br>۰
۹	وقتی موظف هستم که به بیماری بگویم که بیماریش درمانی ندارد و امیدی به زنده ماندنش نیست به سختی می توانم جملات مناسبی انتخاب کنم.	کاملا<br>موافق<br>۱	موافق<br>۲	نه موافق<br>نه مخالف<br>۳	مخالف<br>۴	بشدت<br>مخالف<br>۵	اصلا<br>نظری ندارم<br>۰
۱۰	برایم مهم است که قسمتی از وقت آزاد خود را با بیماران محتضر (درحال مرگ) و بستگان آنها بگذرانم.	کاملا<br>موافق<br>۱	موافق<br>۲	نه موافق<br>نه مخالف<br>۳	مخالف<br>۴	بشدت<br>مخالف<br>۵	اصلا<br>نظری ندارم<br>۰
۱۱	اگر مقابل بیمار در حال مرگ و یا خانواده او گریه کنم، اقدامی غیر حرفه ای انجام داده ام.	کاملا<br>موافق<br>۱	موافق<br>۲	نه موافق<br>نه مخالف<br>۳	مخالف<br>۴	بشدت<br>مخالف<br>۵	اصلا<br>نظری ندارم<br>۰
۱۲	اگر با بیمارانم که در روزهای پایانی عمر خود بسر می برند از نظر احساسی درگیر شوم، باعث خواهد شد که رفتاری غیر حرفه ای مرتکب شوم.	کاملا<br>موافق<br>۱	موافق<br>۲	نه موافق<br>نه مخالف<br>۳	مخالف<br>۴	بشدت<br>مخالف<br>۵	اصلا<br>نظری ندارم<br>۰
۱۳	متأسفانه اگر به مرور با بیمارانم که روزهای پایانی عمر خود را سپری می کنند بیشتر آشنا شوم نهایتاً آسیب خواهم دید.	کاملا<br>موافق<br>۱	موافق<br>۲	نه موافق<br>نه مخالف<br>۳	مخالف<br>۴	بشدت<br>مخالف<br>۵	اصلا<br>نظری ندارم<br>۰
۱۴	بحث با بیمار در مورد تصمیمات پیش از مرگش، برای من بدون استرس می باشد.	کاملا<br>موافق<br>۱	موافق<br>۲	نه موافق<br>نه مخالف<br>۳	مخالف<br>۴	بشدت<br>مخالف<br>۵	اصلا<br>نظری ندارم<br>۰
۱۵	با پنهان کردن احساساتم صحبت با بیمارانی که بزودی فوت خواهند کرد برایم آسانتر می شود.	کاملا<br>موافق<br>۱	موافق<br>۲	نه موافق<br>نه مخالف<br>۳	مخالف<br>۴	بشدت<br>مخالف<br>۵	اصلا<br>نظری ندارم<br>۰
۱۶	زمانیکه که بیمارانی که مدت کوتاهی از عمر آنها باقی مانده است در مورد خودشان با من صحبت می کنند من براحتی به آنها گوش میدهم.	کاملا<br>موافق<br>۱	موافق<br>۲	نه موافق<br>نه مخالف<br>۳	مخالف<br>۴	بشدت<br>مخالف<br>۵	اصلا<br>نظری ندارم<br>۰
۱۷	صرفاً حضور و بودن در کنار بیمارانی که بزودی فوت خواهند کرد تلف کردن وقت است.	کاملا<br>موافق<br>۱	موافق<br>۲	نه موافق<br>نه مخالف<br>۳	مخالف<br>۴	بشدت<br>مخالف<br>۵	اصلا<br>نظری ندارم<br>۰
۱۸	من از صحبت کردن با بیماران در حال فوت اجتناب می کنم.	کاملا<br>موافق<br>۱	موافق<br>۲	نه موافق<br>نه مخالف<br>۳	مخالف<br>۴	بشدت<br>مخالف<br>۵	اصلا<br>نظری ندارم<br>۰
۱۹	در بیمارانی که مدت کوتاهی از عمر طبیعی آنها باقی است در صورت وقوع مشکلات بالقوه کشنده ناشی از عوارض درمان یا روشهای تشخیصی (ایاتروژنیک) علی رغم اعتراض بیمار باید روند احیای بیمار شروع شود.	کاملا<br>موافق<br>۱	موافق<br>۲	نه موافق<br>نه مخالف<br>۳	مخالف<br>۴	بشدت<br>مخالف<br>۵	اصلا<br>نظری ندارم<br>۰

۲۰	اگر من در حال مرگ باشم دوست دارم پزشکم به خواسته های من در مورد تصمیمات پیش و هنگام مرگ من احترام بگذارد.	کاملا موافق ۱	موافق ۲	نه موافق نه مخالف ۳	مخالف ۴	بشدت مخالف ۵	اصلا نظری ندارم ۰
۲۱	دلگرمی دادن به خانواده ای که سوگوار فوت بیمارشان هستند برای من آسان است.	کاملا موافق ۱	موافق ۲	نه موافق نه مخالف ۳	مخالف ۴	بشدت مخالف ۵	اصلا نظری ندارم ۰
۲۲	هنگامیکه به صحبت های بیماری که بزودی فوت خواهد کرد گوش می دهم در حال انجام مداخله درمانی مفیدی هستم.	کاملا موافق ۱	موافق ۲	نه موافق نه مخالف ۳	مخالف ۴	بشدت مخالف ۵	اصلا نظری ندارم ۰
۲۳	من از آن دسته افرادی نیستم که بیمار یا خانواده او را در آغوش بگیرم.	کاملا موافق ۱	موافق ۲	نه موافق نه مخالف ۳	مخالف ۴	بشدت مخالف ۵	اصلا نظری ندارم ۰
۲۴	زمانیکه بیمار و یا خانواده او را در آغوش می گیرم یا دست آنها را می گیرم من یک مداخله درمانی ارائه میدهم.	کاملا موافق ۱	موافق ۲	نه موافق نه مخالف ۳	مخالف ۴	بشدت مخالف ۵	اصلا نظری ندارم ۰
۲۵	وظیفه من به عنوان پزشک توجه به تمام رنجهای بیمار ناشی از مواجهه با مرگ اعّم از تعارضات ذهنی، مذهبی، احساسی، روانی اجتماعی (سایکوسوشیال) و یا فیزیکی می باشد.	کاملا موافق ۱	موافق ۲	نه موافق نه مخالف ۳	مخالف ۴	بشدت مخالف ۵	اصلا نظری ندارم ۰
۲۶	اجازه دادن به اینکه شرایط قابل درمان ساده ای نظیر پنومونی یا سپسیس بیمار محتضر (در حال مرگ) را از پای درآورد معادل کمک به خودکشی او می باشد.	کاملا موافق ۱	موافق ۲	نه موافق نه مخالف ۳	مخالف ۴	بشدت مخالف ۵	اصلا نظری ندارم ۰
۲۷	من به اندازه سایر بیماران با بیمار محتضر (در حال مرگ) راحت هستم.	کاملا موافق ۱	موافق ۲	نه موافق نه مخالف ۳	مخالف ۴	بشدت مخالف ۵	اصلا نظری ندارم ۰
۲۸	درمان بیمار محتضر (در حال مرگ) یکی از ناخوشایندترین وجهه های شغل من است.	کاملا موافق ۱	موافق ۲	نه موافق نه مخالف ۳	مخالف ۴	بشدت مخالف ۵	اصلا نظری ندارم ۰
۲۹	درصورتیکه امکان پذیر باشد من از بیماری که بعلت یک وضعیت غیر قابل برگشت در حال مرگ می باشد دوری میکنم.	کاملا موافق ۱	موافق ۲	نه موافق نه مخالف ۳	مخالف ۴	بشدت مخالف ۵	اصلا نظری ندارم ۰
۳۰	من از اینکه به بیمار مستقیما بگویم که او در حال مرگ می باشد تا حد امکان اجتناب می کنم.	کاملا موافق ۱	موافق ۲	نه موافق نه مخالف ۳	مخالف ۴	بشدت مخالف ۵	اصلا نظری ندارم ۰
۳۱	کنار آمدن با خانواده بیمار در حال مرگ از کنار آمدن با خانواده بقیه بیماران برای من مشکل تر است.	کاملا موافق ۱	موافق ۲	نه موافق نه مخالف ۳	مخالف ۴	بشدت مخالف ۵	اصلا نظری ندارم ۰
